# Supplementary figures and images for: Comprehensive structural characterization of the human AAA+ disaggregase CLPB in the apo- and substrate-bound states reveals a unique mode of action driven by oligomerization
Source: PLoS Biol. 2023 Feb 6;21(2):e3001987. doi: 10.1371/journal.pbio.3001987 (PMC9934407; doi:10.1371/journal.pbio.3001987)

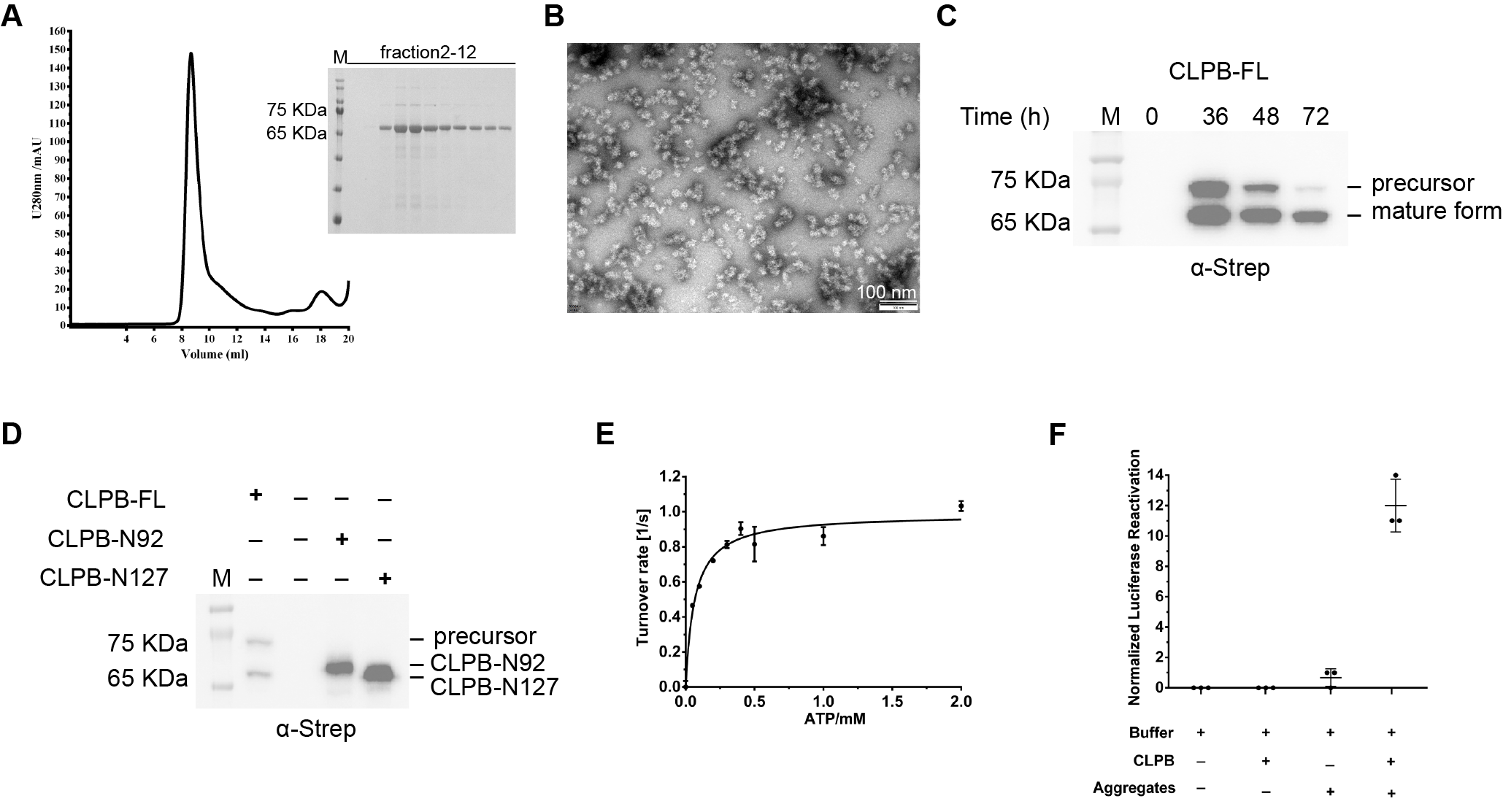

Supplement: S1 Fig — (A) The purification of CLPB-N92, analyzing by size-exclusion chromatography (left) and SDS-PAGE (right). (B) Negative staining electron microscopy of the peak fraction in (A). Result shows that CLPB-N92 formed large aggregates and was highly heterogenous in size. (C) Time course of the C-terminal Step-tagged full-length CLPB (CLPB-FL) expression in transiently transfected HEK-293T cells. (D) CLPB-FL, CLPB-N92, and CLPB-N127 expression in transiently transfected HEK-293T cells. Data show that the CLPB-N127 has the same molecular weight as the mature form of CLPB. (E) ATPase activity of CLPB-N127 (S1 Data). (F) Disaggregase activity of CLPB-N127 (S1 Data). (TIF) [file pbio.3001987.s001.tif]

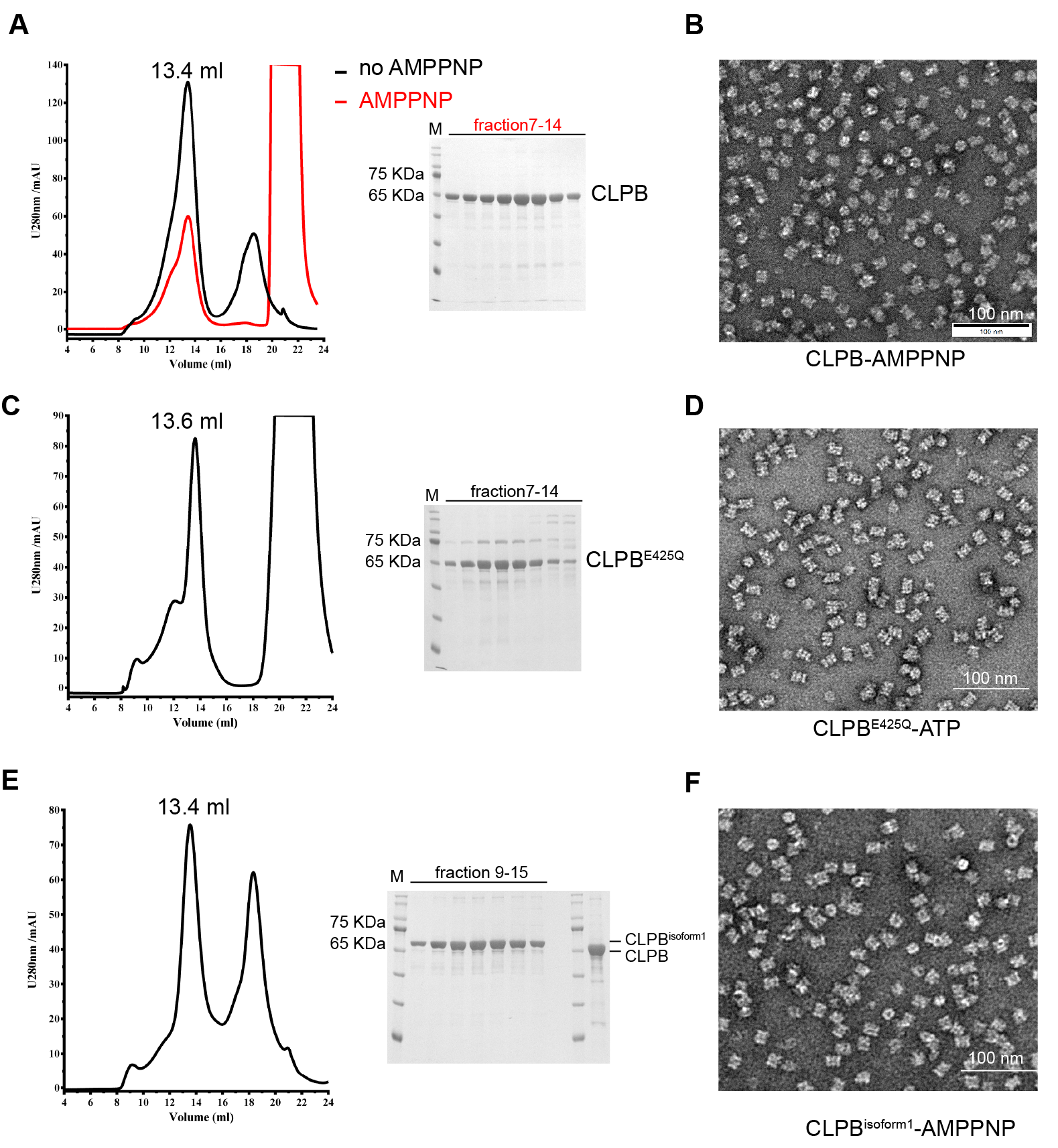

Supplement: S2 Fig — (A) Purification of CLPB using size-exclusion chromatography with AMPPNP (red line) or without AMPPNP (black line). Corresponding fractions were analyzed by SDS-PAGE (right panel). (B) Representative nsEM image of the peak fraction in (A). (C) Purification of CLPBE425Q using size-exclusion chromatography in the presence of ATP. Corresponding fractions were analyzed by SDS-PAGE (right panel). (D) Representative nsEM image of the peak fraction in (C). (E) Purification of CLPBisodorm1 using size-exclusion chromatography. Corresponding fractions were analyzed by SDS-PAGE (right panel). (F) Representative nsEM image of the peak fraction in (E). (TIF) [file pbio.3001987.s002.tif]

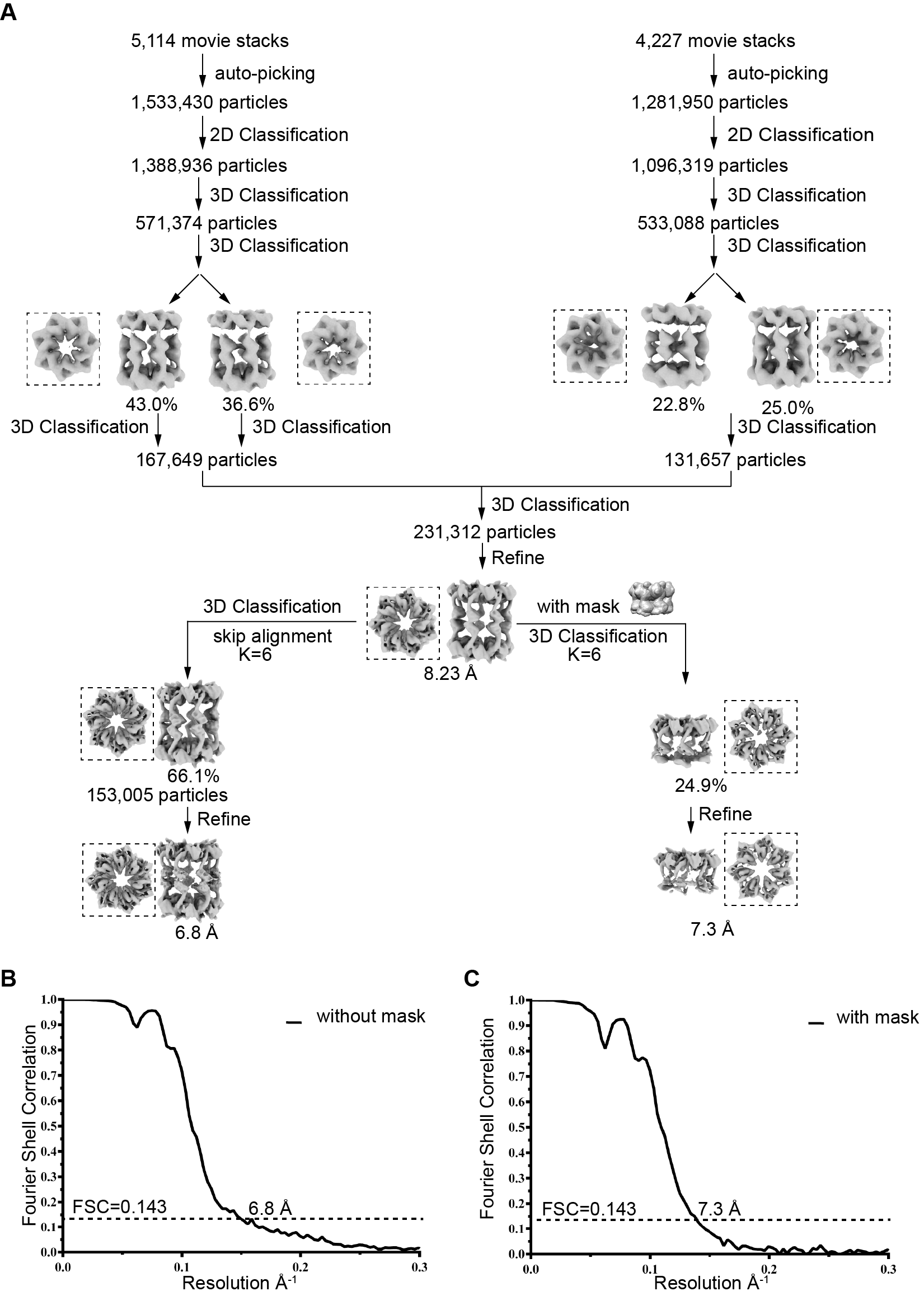

Supplement: S3 Fig — (A) Image processing workflow of the CLPB dataset (see Methods for details). (B, C) Fourier shell correlation (FSC) curves for the final cryo-EM map of the double-heptameric complex (B) or heptameric complex (C) using the gold standard FSC 0.143 criteria. (TIF) [file pbio.3001987.s003.tif]

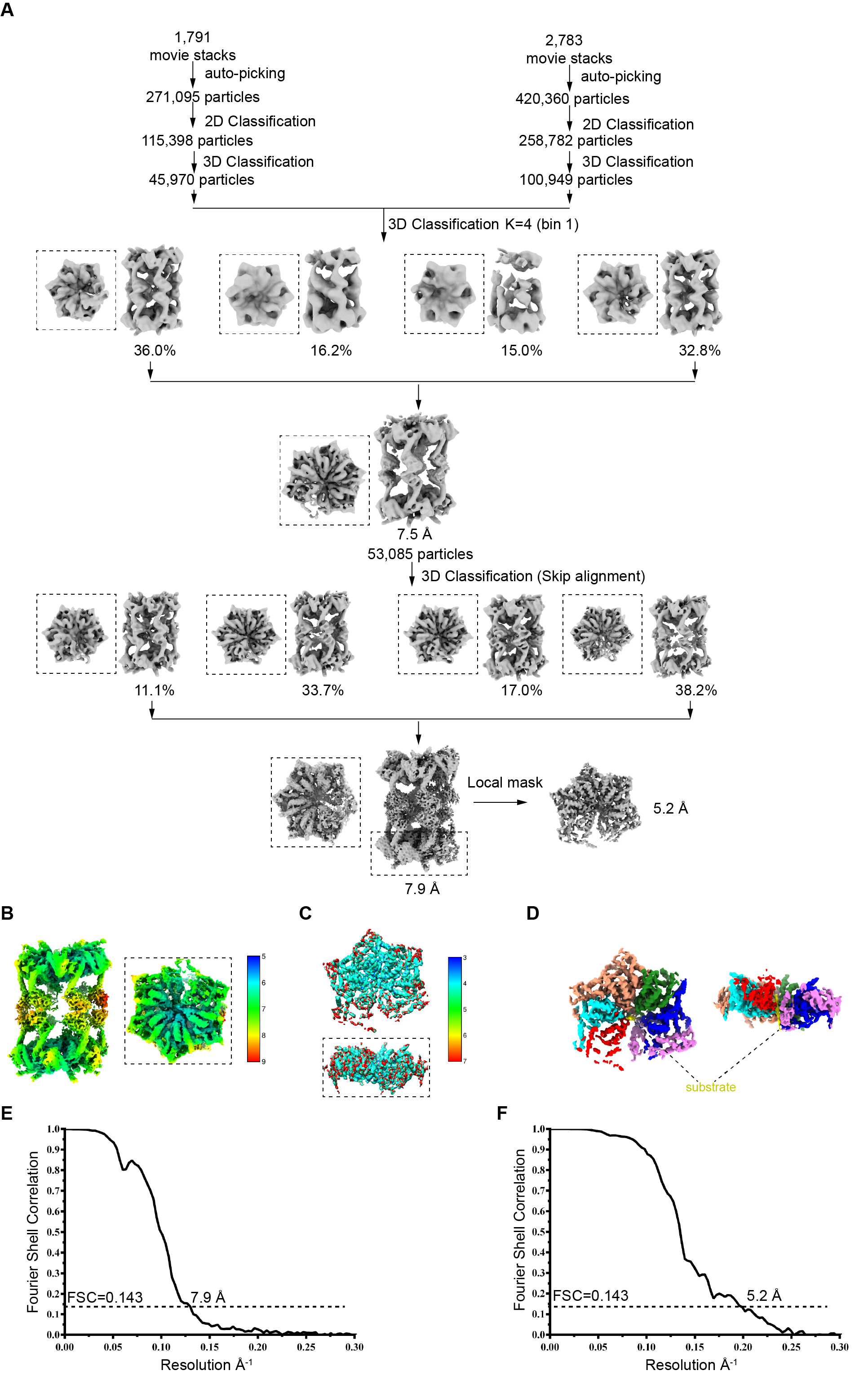

Supplement: S4 Fig — (A) Image processing workflow of the CLPBE425Q dataset. (B, C) Local resolution estimation of CLPBE425Q double-hexamer (B) or NBD alone (C). (D) Density map of the NBD alone. The central substrate is colored yellow. (E, F) Fourier shell correlation curve of the final map of the CLPBE425Q double-hexamer (E) and NBD (F). (TIF) [file pbio.3001987.s004.tif]

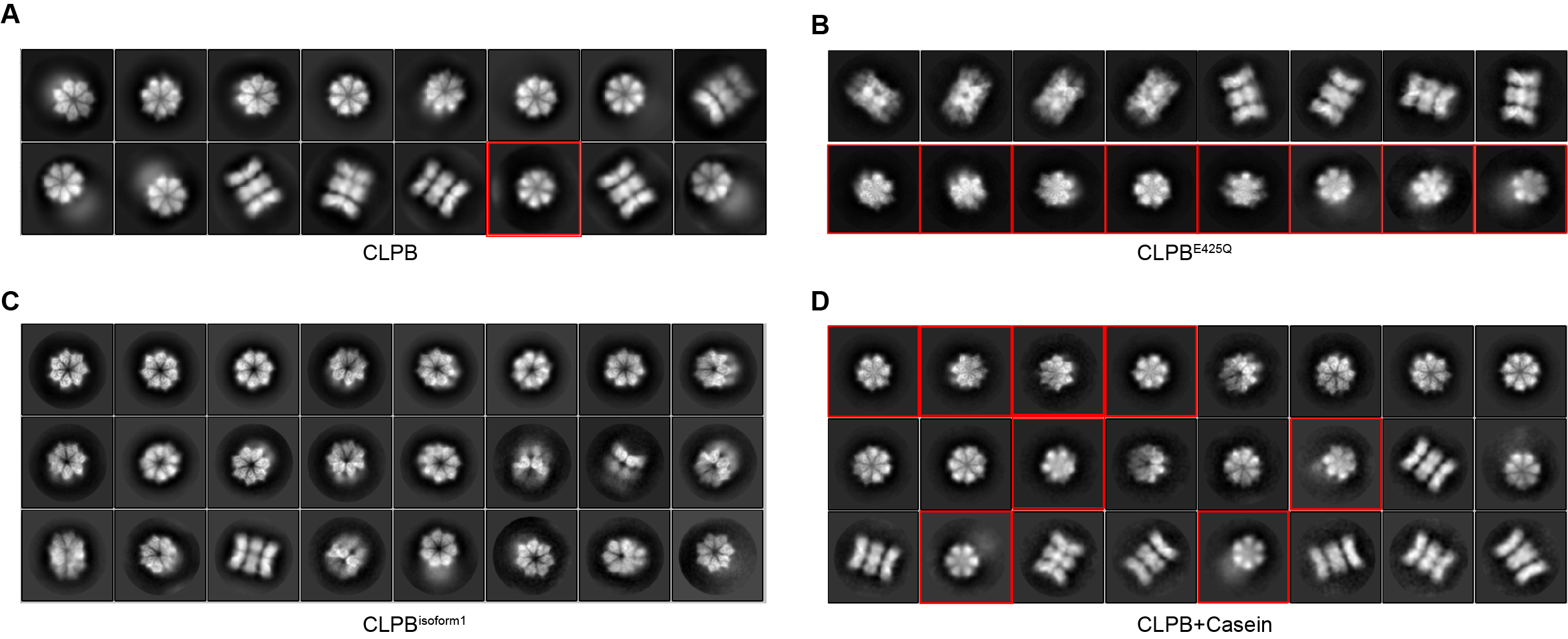

Supplement: S5 Fig — (A–D) Representative 2D classification averages of CLPB (A), CLPBE425Q (B), CLPBisoform1 (C), and CLPB+Casein (D) datasets. The top views with hexameric features are indicated by red boxes. (TIF) [file pbio.3001987.s005.tif]

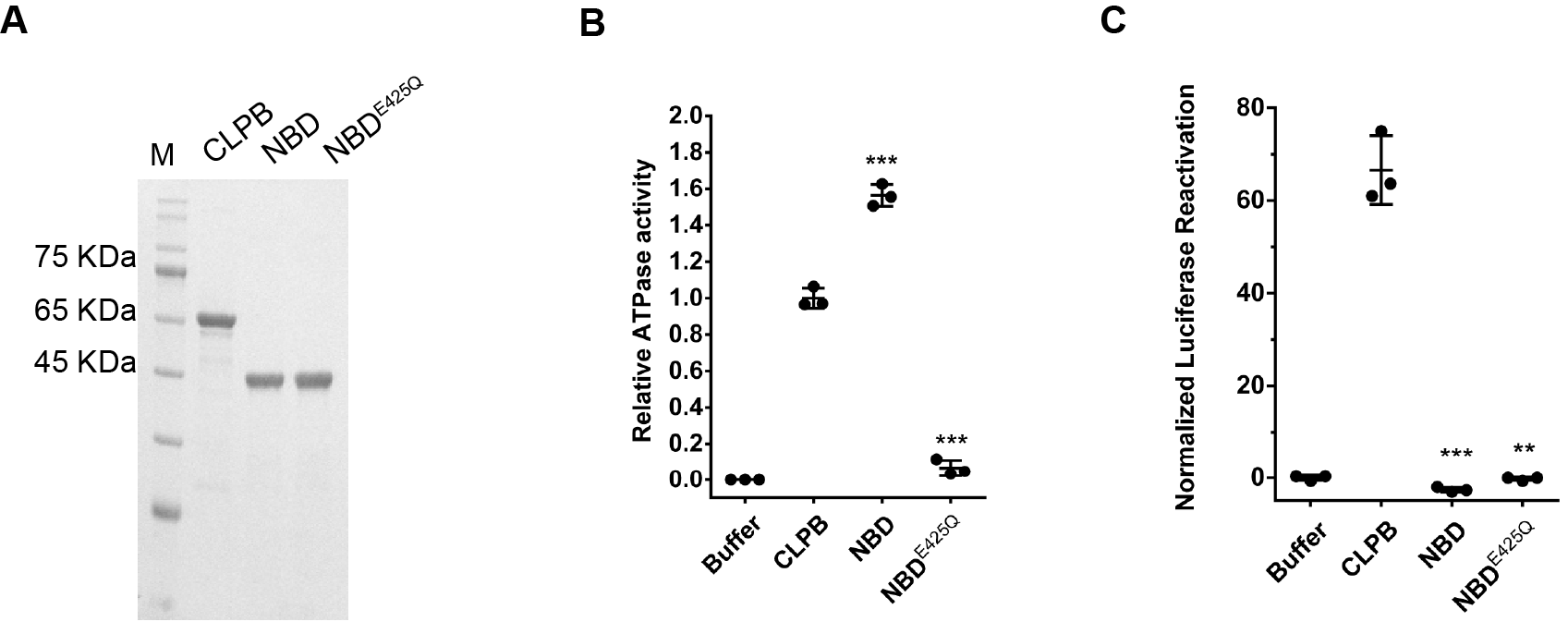

Supplement: S6 Fig — (A) SDS-PAGE analysis of the purified proteins. (B) ATPase assays of CLPB, NBD, and NBDE425Q. Results show that NBDE425Q has nearly no ATPase activity. NBD has strong ATPase activity. ATPase activity was compared to CLPB (N = 3, individual data points shown as dots, bars show mean ± SD, *p < 0.05, **p < 0.01, ***p < 0.0001) (S3 Data). (C) Disaggregase activity assay of CLPB, NBD, and NBDE425Q. The results show that NBD and NBDE425Q abolish the disaggregase activity of CLPB. Disaggregase activity was compared to CLPB (N = 3, individual data points shown as dots, bars show mean ± SD, **p < 0.01, ***p < 0.0001) (S3 Data). (TIF) [file pbio.3001987.s006.tif]

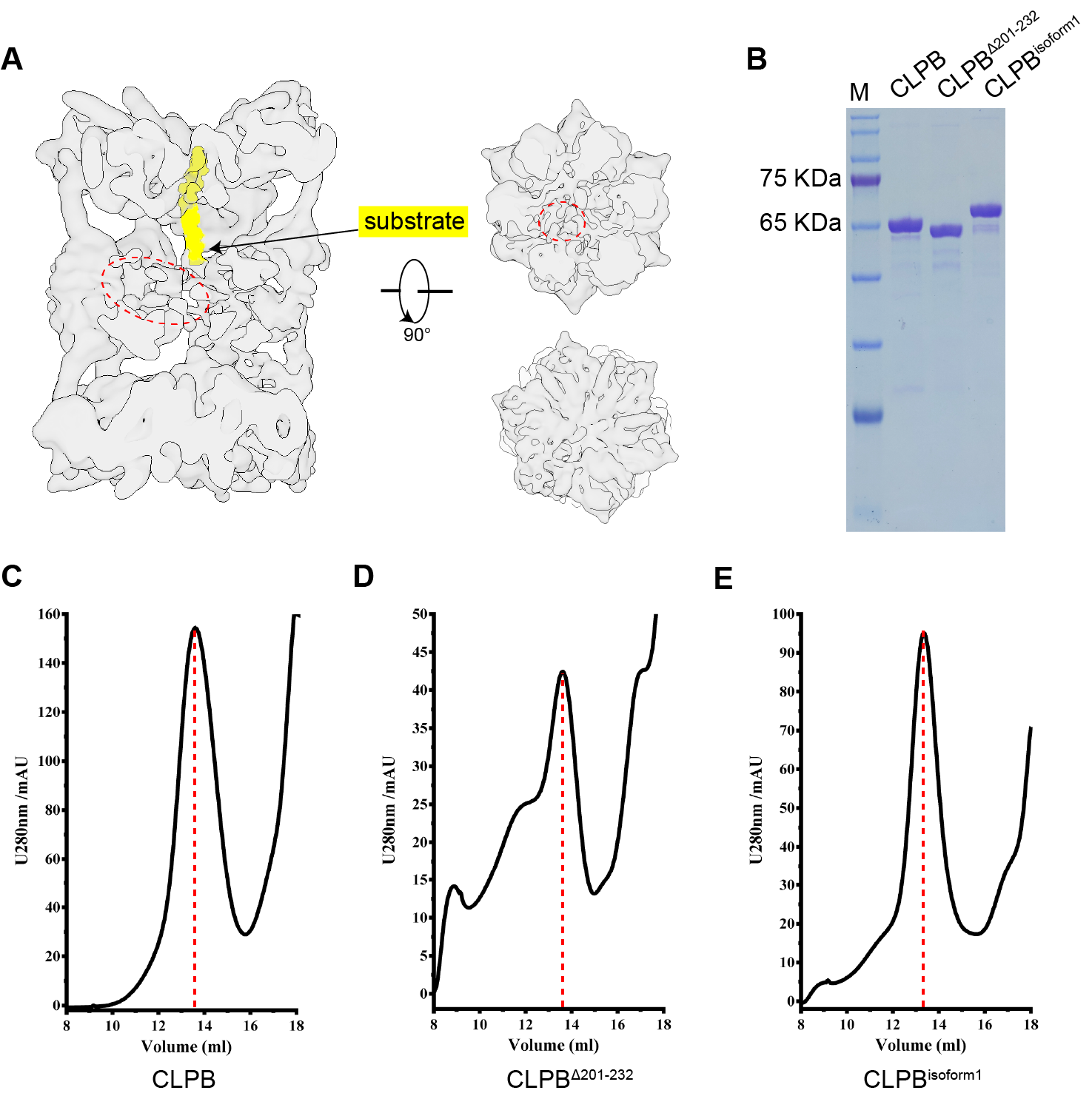

Supplement: S7 Fig — (A) Residual densities around the ANK domains, extending towards the central channel of the CLPBE425Q complex in the substrate-bound state. The extra densities are highlighted by a dotted ellipse. The density of the substrate is shown in yellow. (B) SDS-PAGE analysis of the purified proteins. (C–E) Purification of CLPB, CLPBΔ201–232, and CLPBisoform1 complexes using size-exclusion chromatography. (TIF) [file pbio.3001987.s007.tif]

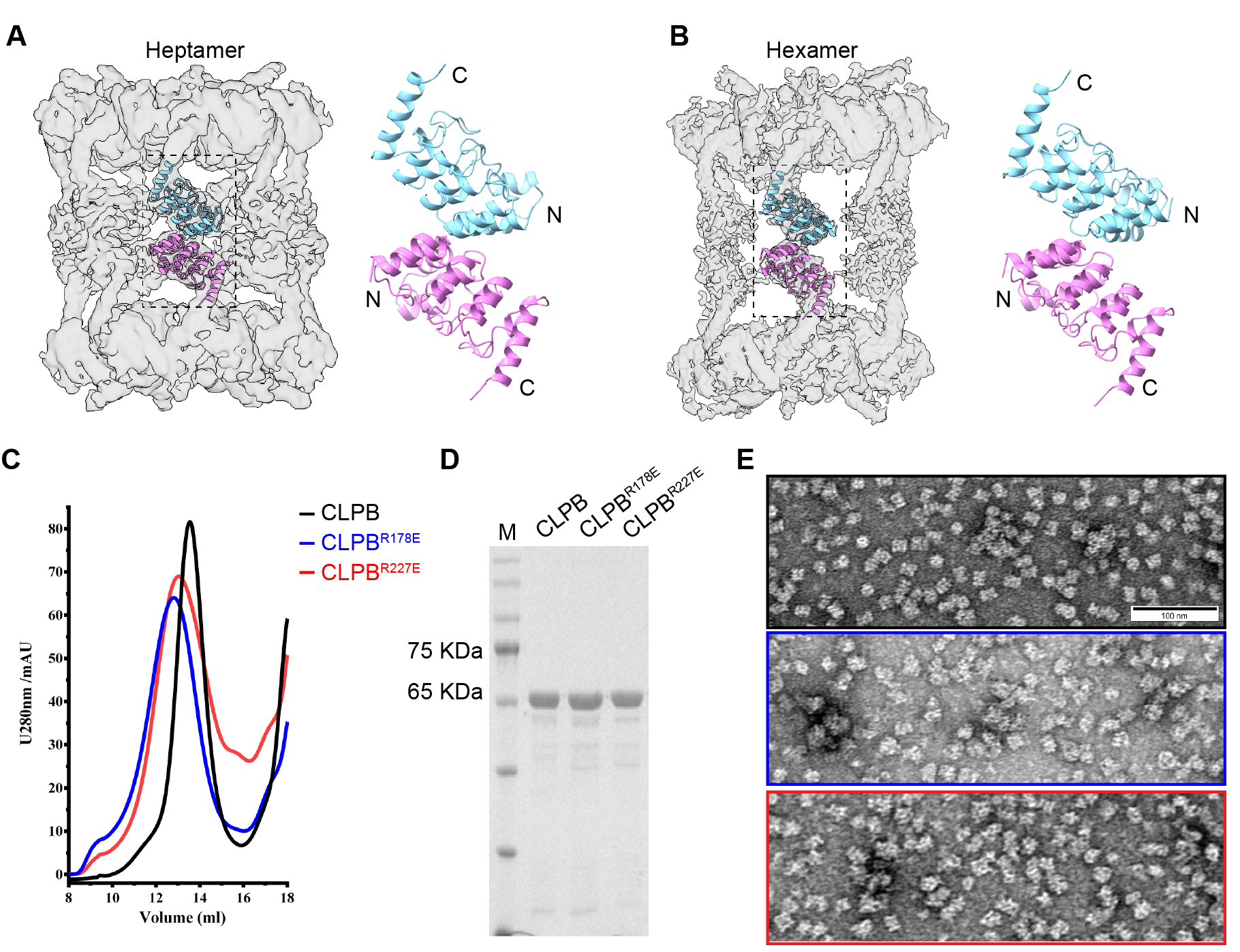

Supplement: S8 Fig — (A, B) Rigid-body fitting of the crystal structure of the ANK domain into the density maps of the double-heptamer (A) and double-hexamer (B). (C) Purification of the CLPB, CLPBR178E, and CLPBR227E complexes using size-exclusion chromatography. (D) SDS-PAGE analysis of the purified proteins. (E) Representative nsEM images of the CLPB (black line rectangle), CLPBR178E (blue line rectangle), and CLPBR227E complexes (red line rectangle). (TIF) [file pbio.3001987.s008.tif]

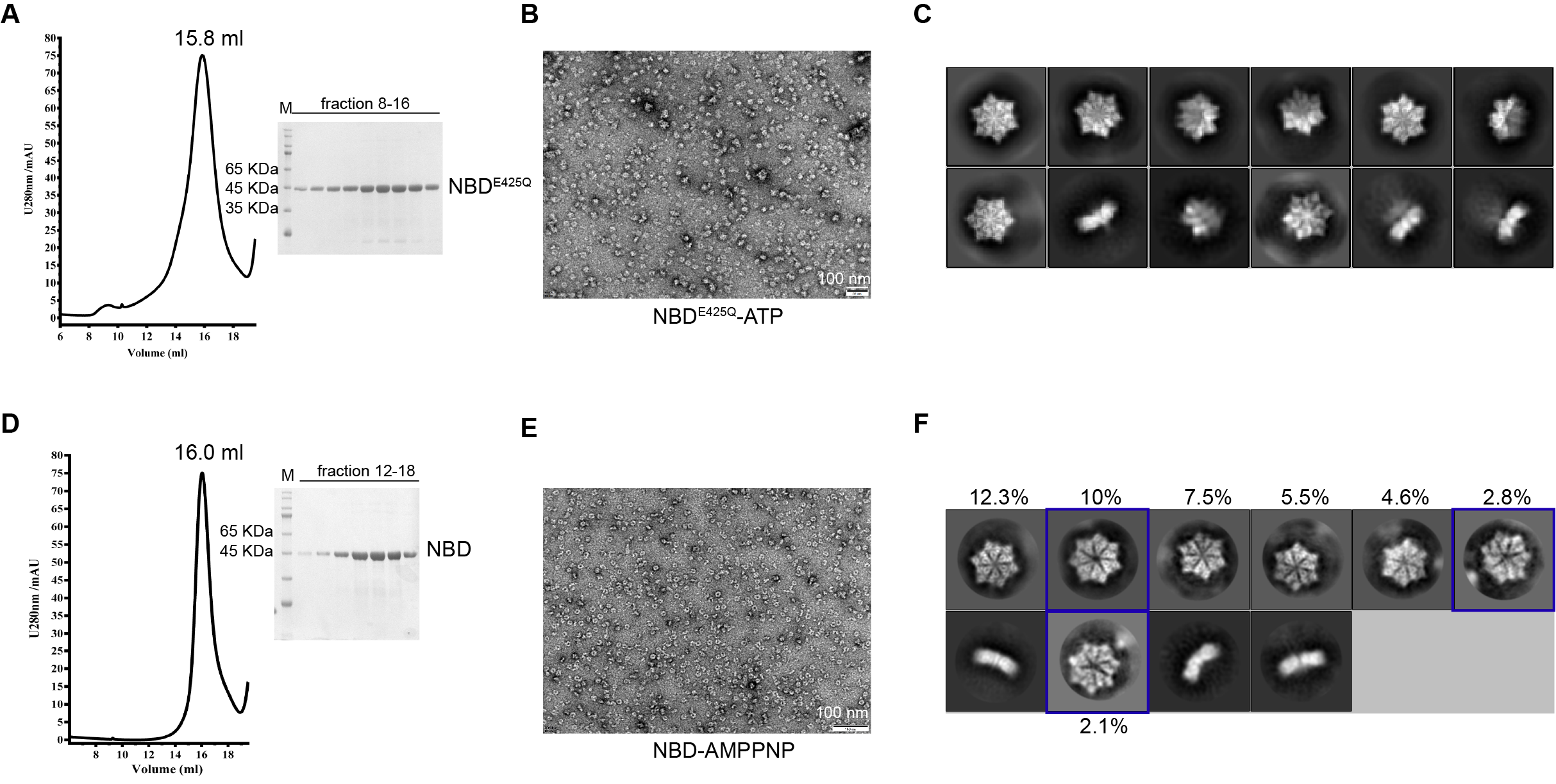

Supplement: S9 Fig — (A) Purification of NBDE425Q using size-exclusion chromatography in the presence of ATP. Corresponding fractions were analyzed by SDS-PAGE. (B, C) Representative nsEM image (B) and 2D classification averages of cryo-EM dataset (C) of the peak fraction in (A). (D) Purification of NBD using size-exclusion chromatography in the presence of AMPPNP. Corresponding fractions were analyzed by SDS-PAGE (right panel). (E, F) Representative nsEM image (E) and 2D classification averages cryo-EM dataset (F) of the peak fraction in (D). The hexameric ring is more compact than heptameric ring, and the diameter of central pore of hexamer is much smaller than that of heptamer. (TIF) [file pbio.3001987.s009.tif]

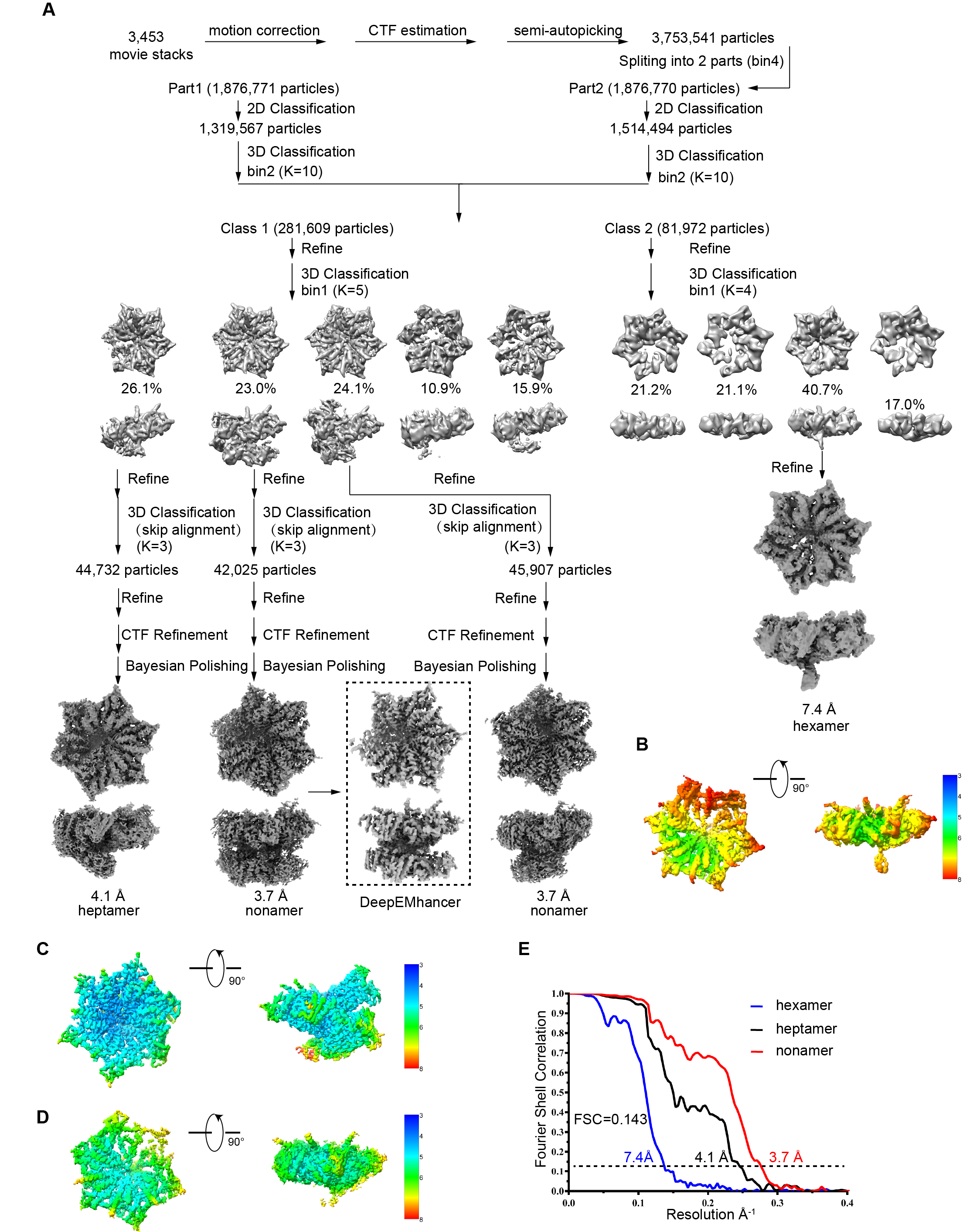

Supplement: S10 Fig — (A) Image processing workflow of the NBDE425Q dataset. The micrographs were subjected to motion correction and CTF estimation. The auto-picked particles were subjected to multiple rounds of 2D and 3D classifications. Three different oligomeric arrangements, hexameric, heptameric, and nonameric were identified. (B–D) Local resolution estimation of the density maps of the hexamer (B), heptamer (C), and nonamer (D) in (A). (E) Fourier shell correlation (FSC) curves of the final cryo-EM maps of hexamer (blue line), heptamer (black line), and nonamer (red line), using the gold standard FSC 0.143 criteria. (TIF) [file pbio.3001987.s010.tif]

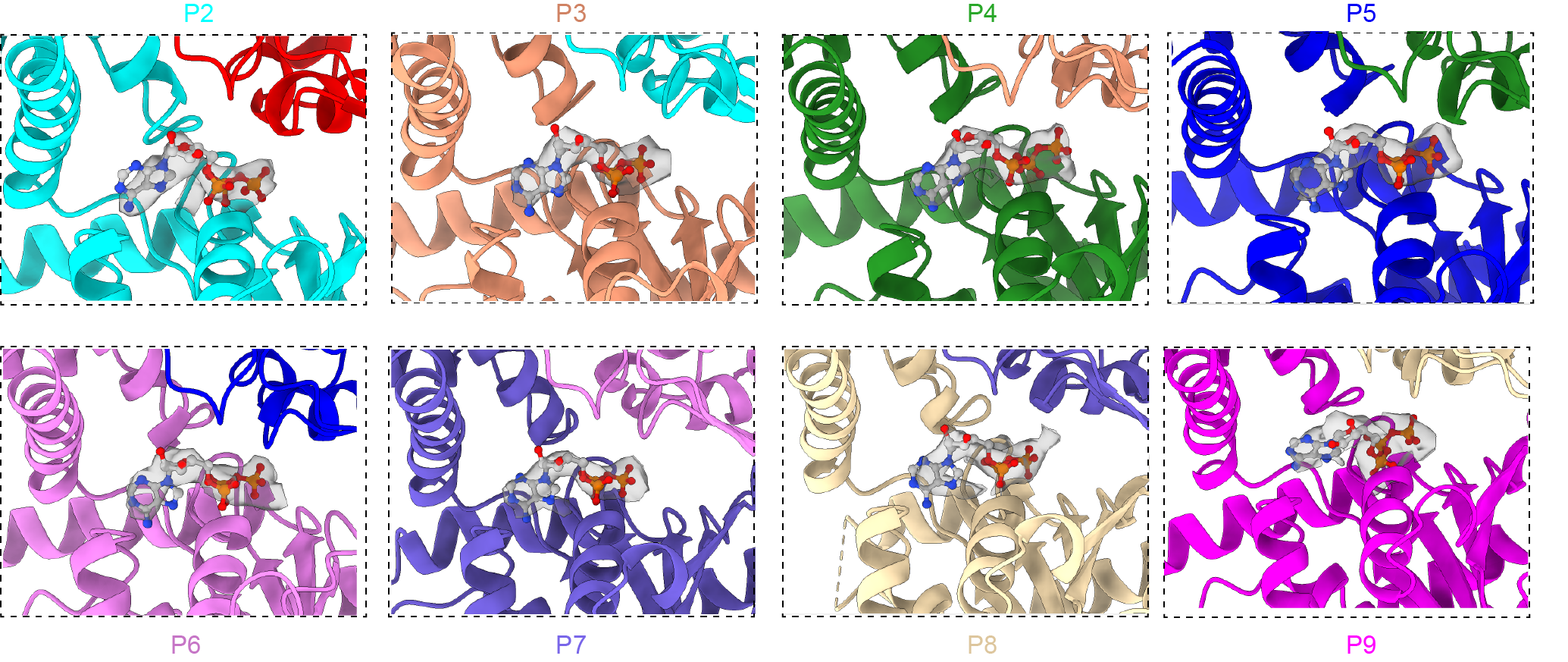

Supplement: S11 Fig — All the 8 ATPase sites in the nonamer are occupied by ATP. The atomic models are color-coded for different protomers. The segmented density maps of ATP were superimposed with the atomic model. (TIF) [file pbio.3001987.s011.tif]
