# Supplementary material for: Comprehensive structural characterization of the human AAA+ disaggregase CLPB in the apo- and substrate-bound states reveals a unique mode of action driven by oligomerization
Source: PLoS Biol. 2023 Feb 6;21(2):e3001987. doi: 10.1371/journal.pbio.3001987 (PMC9934407; doi:10.1371/journal.pbio.3001987)
Supplement: S1 Table — (DOCX) [file pbio.3001987.s012.docx]

**S1 Table.** **Cryo-EM data collection, refinement and validation statistics**

|  | CLPB  EMD-33105 | CLPB^E425Q^  EMD-33106 | Hexamer  EMD-33109 | NBD^E425Q^  Heptamer  EMD-33110 | Nonamer  EMD-33104  PDB: 7XBK |
| --- | --- | --- | --- | --- | --- |
| **Data collection and processing** |  |  |  |  |  |
| Nominal magnification | 81,000X | 36,000X | 64,000X | | |
| Voltage (kV) | 300 | 300 | 300 | | |
| Electron exposure (e^–^/Å^2^) | 60 | 58 | 50 | | |
| Defocus range (μm) | -1 to -1.4 | -1.2 to -1.8 | -1 to -1.4 | | |
| Pixel size (Å) | 1.07 | 1.052 | 1.08 | | |
| Micrographs | 9,341 | 4,574 | 3,453 | | |
| Symmetry imposed | C1 | C1 | C1 | | |
| Map resolution (total) (Å) | 6.8 | 7.9 | 7.4 | 4.1 | 3.7 |
| FSC threshold | 0.143 | 0.143 | 0.143 | 0.143 | 0.143 |
| **Refinement**  Map sharpening *B* factor (Å^2^) | -89.9 | -155.0 | -237.2 | -83.4 | -97.3 |
| Model composition  Non-hydrogen atoms  Protein residues | -  - | -  - | -  - | -  - | 25,745  3,156 |
| Ligands  *B* factor (Å^2^)  Protein  Ligands | -  -  - | -  -  - | -  -  - | -  -  - | 8 Mg^2+^, 8ATP  88.42  66.90 |
| R.m.s. deviations  Bond lengths (Å)  Bond angles (°) | -  - | -  - | -  - | -  - | 0.005  0.863 |
| Validation  MolProbity score  Clashscore  Poor rotamers (%) | -  -  - | -  -  - | -  -  - | -  -  - | 1.48  5.67  0.00 |
| Ramachandran plot  Favored (%)  Allowed (%)  Disallowed (%) | -  -  - | -  -  - | -  -  - | -  -  - | 97.04  2.93  0.03 |
