# Supplementary material for: Comprehensive structural characterization of the human AAA+ disaggregase CLPB in the apo- and substrate-bound states reveals a unique mode of action driven by oligomerization
Source: PLoS Biol. 2023 Feb 6;21(2):e3001987. doi: 10.1371/journal.pbio.3001987 (PMC9934407; doi:10.1371/journal.pbio.3001987)
Supplement: S2 Table — (DOCX) [file pbio.3001987.s013.docx]

**S2 Table.** **Data collection and refinement statistics of the CLPB_ANK**

|  | CLPB_ANK |
| --- | --- |
| **Data collection**^a^ |  |
| Space group | C121 |
| Cell dimensions |  |
| *a*, *b*, *c* (Å) | 30.31 67.68 84.98 |
| α, β, γ | 90.0 96.05 90.00 |
| Resolution (Å) | 33.84-1.99 (2.04-1.99)^b^ |
| *R*_merge_ | 0.058 (0.623) |
| *R_meas_* | 0.070 (0.739) |
| *I* / σ*I* | 11.2 (1.9) |
| *CC(1/2)* | 0.998 (0.865) |
| Completeness (%) | 99.4 (99.5) |
| Redundancy | 1.6 (1.7) |
| **Refinement** |  |
| Resolution (Å) | 2.1 |
| No. reflections | 9,966 |
| *R*_work_/*R*_free_ (%) | 20.6/23.6 |
| No. atoms |  |
| Protein | 1,287 |
| Water | 29 |
| *B*-factors (Å^2^) |  |
| Protein | 49.0 |
| Water | 43.9 |
| R.m.s. deviations |  |
| Bond lengths (Å) | 0.003 |
| Bond angles (°) | 0.629 |
| Ramachandran stat.(%) | 98.78 / 1.22 / 0.0 / 0.0^c^ |

^a^ One crystal was used for data collection.

^b^ Values in parentheses are for the highest-resolution shell.

^c^ Values are in percentage and are for most favoured, additionally allowed, generously allowed, and disallowed regions in Ramachandran plots, respectively.
