# Supplementary material for: Comprehensive structural characterization of the human AAA+ disaggregase CLPB in the apo- and substrate-bound states reveals a unique mode of action driven by oligomerization
Source: PLoS Biol. 2023 Feb 6;21(2):e3001987. doi: 10.1371/journal.pbio.3001987 (PMC9934407; doi:10.1371/journal.pbio.3001987)

Figure 6A

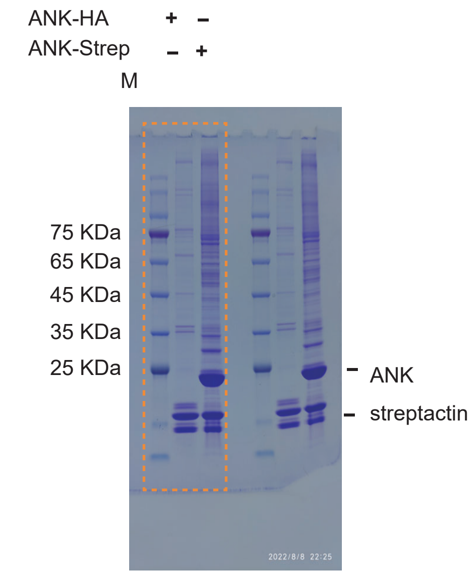

Figure 6D

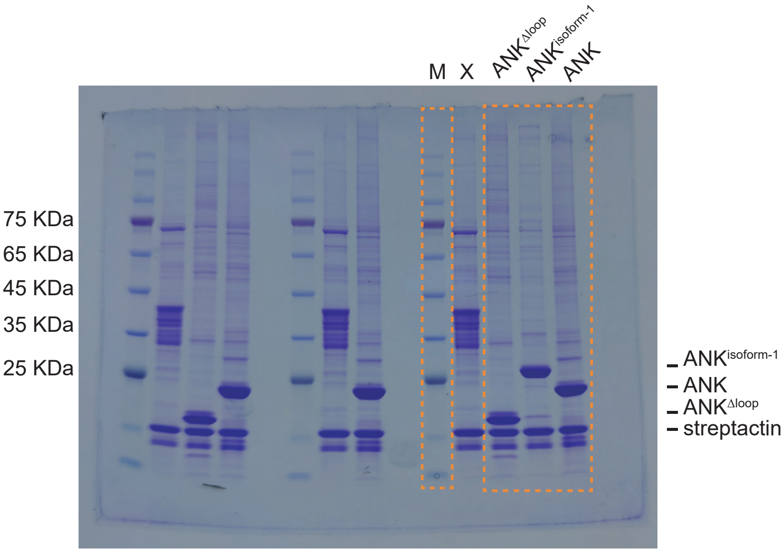

Figure S1A

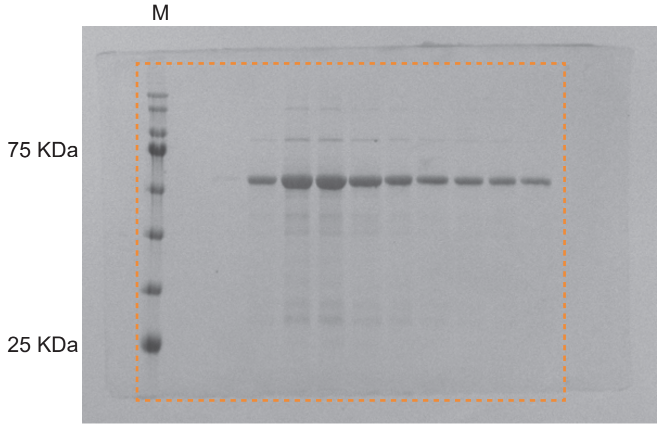

Figure S1C

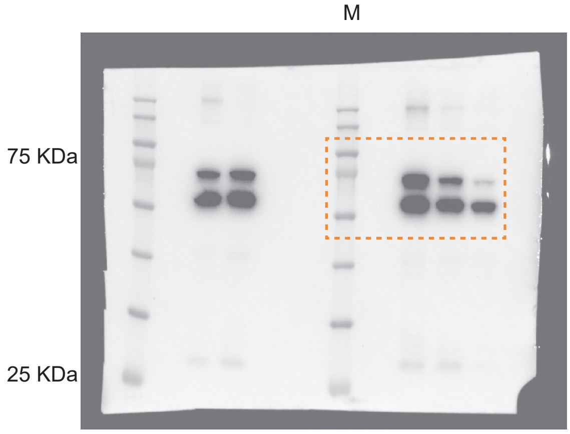

Figure S1D

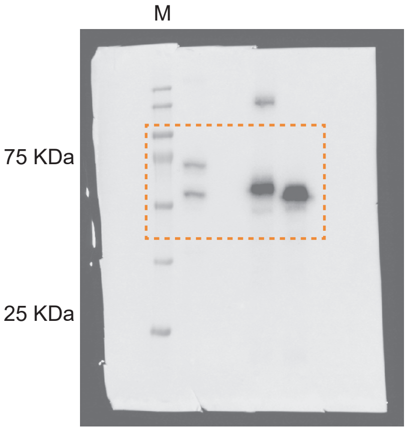

Figure S2A

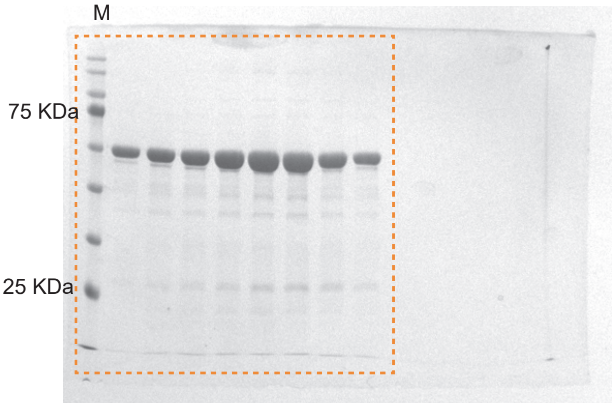

Figure S2C

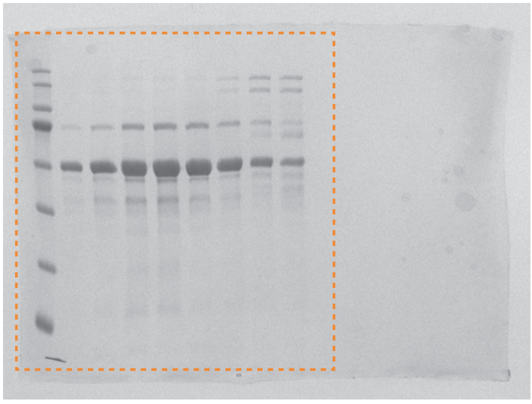

Figure S2E

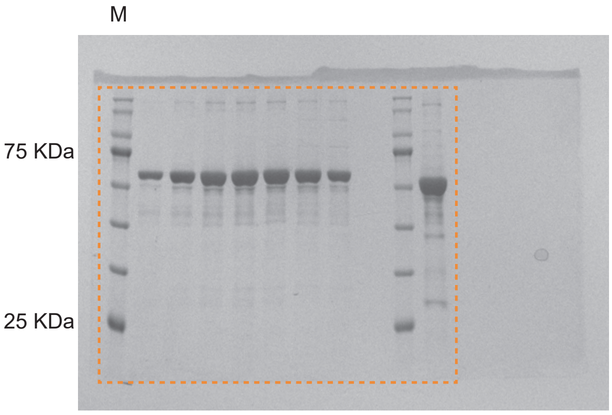

Figure S6A

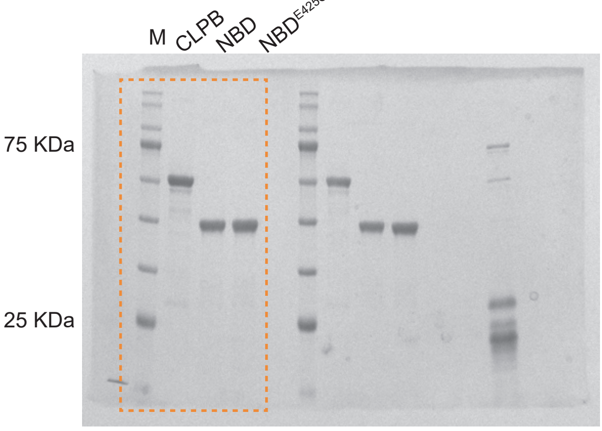

Figure S7B

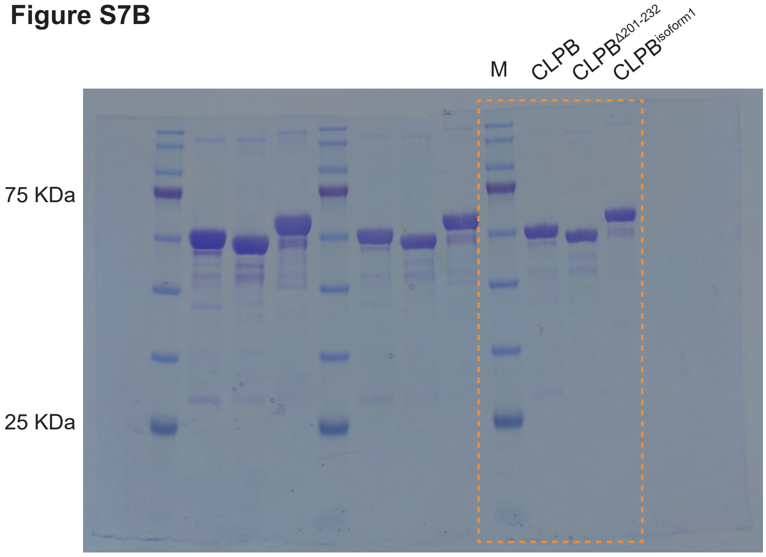

Figure S9A

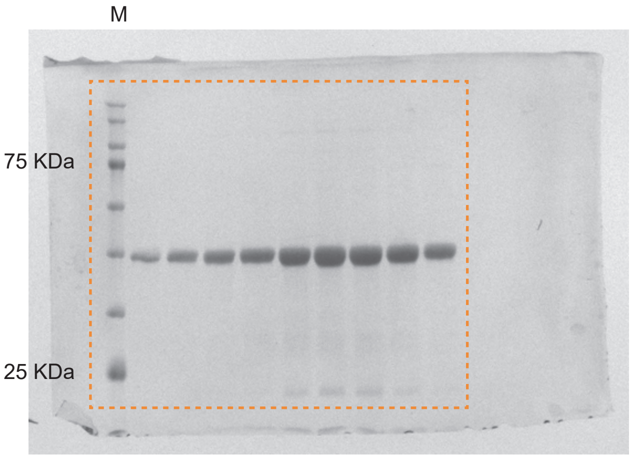

Figure S8D

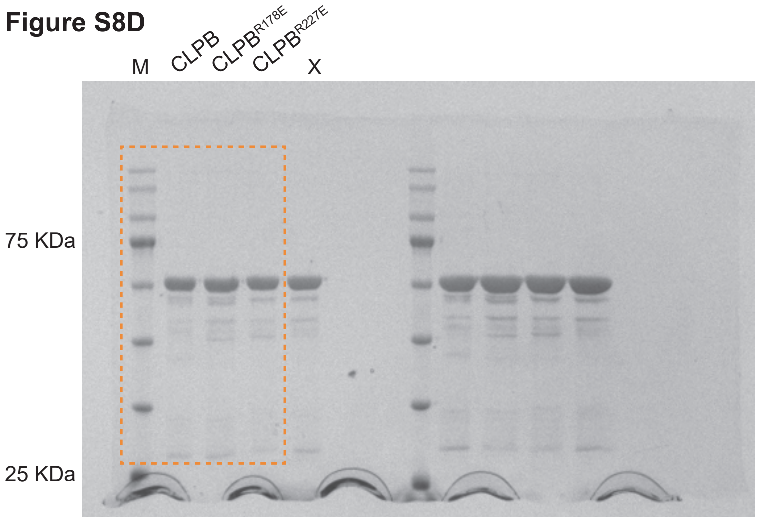

Figure S9D

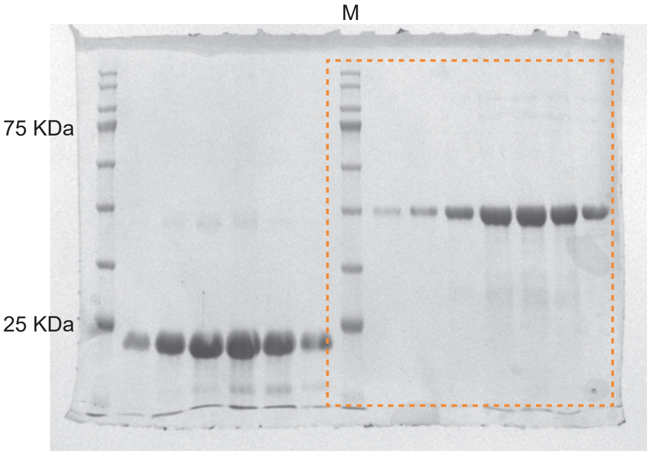

Supplement: S1 Raw image — (PDF) [file pbio.3001987.s016.pdf]
