# Supplementary material for: Comprehensive structural characterization of the human AAA+ disaggregase CLPB in the apo- and substrate-bound states reveals a unique mode of action driven by oligomerization
Source: PLoS Biol. 2023 Feb 6;21(2):e3001987. doi: 10.1371/journal.pbio.3001987 (PMC9934407; doi:10.1371/journal.pbio.3001987)
Supplement: Attachment — Submitted filename: Reviews-V7.pdf [file pbio.3001987.s024.pdf]

We sincerely thank all the reviewers for their encouraging and constructive comments, which are very helpful for us to prepare the revision.

Reviewer #1:

The human mitochondrial AAA+ ATPase CLPB (aka, HSP78 and Skd3) is attracted attentions because it can mediate disaggregation of various proteins and also is related with various diseases. Compared with canonical AAA+, is unique in the N terminal ankyrin repeat domain (ANK). The manuscript describes the cryoEM structures of CLPB and studied the role of the ANK domain. The authors found that CLPB assembled into homo-tetradecamers in apo-state and changed to homo-dodecamers upon binding to substrates. As the ANK domain located at the interface of heptamer or hexamer, the authors concluded that the ANK domain is responsible for the higher-order assembly. The authors also suggested that the ANK domain is substrate binding site from the results of MS analysis of ANK interactomes. The putative disaggregases, Hsp104 or ClpB, contains two NBD in a subunit. Thus it seems to be reasonable that CLPB with one NBD exists as double ring complex to take similar configuration. The experiments were well designed and the results are persuasive. Therefore, the manuscript is valid for publication.

However, I recommend the authors to consider and discuss a couple of following problems raised from the results.

The transition from tetradecamer to dodecamer requires disassembly of the complex. Thus, CLPB should take dynamic oligomeric structural change, possible with the dissociation to monomers. The relatively low resolution should be due to the dynamic structural change.

This is an important point. In the presence of a model substrate casein, around 50% of the tetradecamers could be converted to dodecamers (Figure 2C), indicating a high propensity of CLPB complex to undergo large structural remodeling. During image processing, we have also tried “symmetry expansion”, “density subtraction” and “masked-based local refinement” for the tetradecamer and dodecamer particles. But all of these efforts were not effective in further improving the map resolutions. The dodecamer is slightly better than the tetradecamer: as shown in S4 Figure, α-helices were resolved with side chain bumps discernible at 5-Å resolution.

We think that several factors contribute to the conformational flexibility of the full-length complexes. The first is the hexamer/heptamer interface. Although the ANK domains from the two heptamer/hexamers interact with each other, they do not have lateral interactions. Thus, the interface between two heptamers/hexamers is rather flexible. The second is the flexibility within each heptamers/hexamers, because the AAA+ domain rings are known to adopt unsymmetrical and dynamic conformations.

We have taken this suggestion and added this discussion in the revision (Page7, line 139 to 145).

The ANK domain exists in the interface between the rings. Thus, it seems to be difficult to contribute to the interaction with substrates. I think the interactome analysis of the ANK domain might be artefact.

I think that the manuscript is acceptable after the revision according to the above problems.

As shown in Figures 1 and 3, although the ANK domain mediates the formation of the double-hexamer/heptamer, it has no lateral interaction with the adjacent ANK domains within the hexamer/heptamer. The ANK domain is connected to the NBD through a single α-helix, and there is a large empty space between these link helices. Thus, the chamber enclosed by two layers of ANK domains is freely accessible from the solvent. The protrusions of the protein aggregates could gain access to the inner surface of the ANK domain (including the insertion loop) through the empty space between lateral ANK domains.

In addition, the ANK domain only uses a small surface area for inter-ANK interactions. Most of the surfaces are fully exposed. It remains possible that some of these areas may also be used for substrate interaction.

In the revision, we have performed additional MS experiments. The results and discussions on the possible role of the ANK domain in substrate binding have been updated (Page11-12).

Reviewer #2:

In this study, Wu et al. present a structural characterization of the human HSP100 family protein CLPB. Although diverse conformations of the purified protein limited the resolution, the authors used rigid body fitting to model the CLPB structure. Interestingly, the authors found that CLPB assembles into homo-tetradecamers in the apo-state which are converted into dodecamers upon substrate binding. The study confirms the importance of ANK domain for the substrate recognition and suggests its importance for higher-order organization (double hexameric structure) that is critical for disaggregase cycle of CLPB.

This is interesting study which provides novel and unexpected insights into the structural organization of CLPB and which allows mapping and functional interpretation of disease-related mutations and understanding their possible implications on the CLPB function.

However, the authors should consider the following points:

1.The authors refer to substrate density in the central channel of CLPB hexamers in Fig. 1D. However, it remains unclear how a substrate density is distinguished from conformational changes in CLPB that are induced by the Walker B mutation (impairing ATP hydrolysis)?

As we have described in the Method, with a mask-based refinement, the NBD ring of the dodecamer could be improved to 5.2-Å resolution (Rebuttal Figure 1). In this map, the secondary structural features of the NBDs are well resolved, a polypeptide within the central channel of the NBD ring could be identified. In the revision, we have added these panels to S4D Figure.

In Figure 1D, to highlight the overall structure of the double-hexamer, the global density map (low-pass filtered) was used for figure preparation. Therefore, it appears to be of very low resolution.

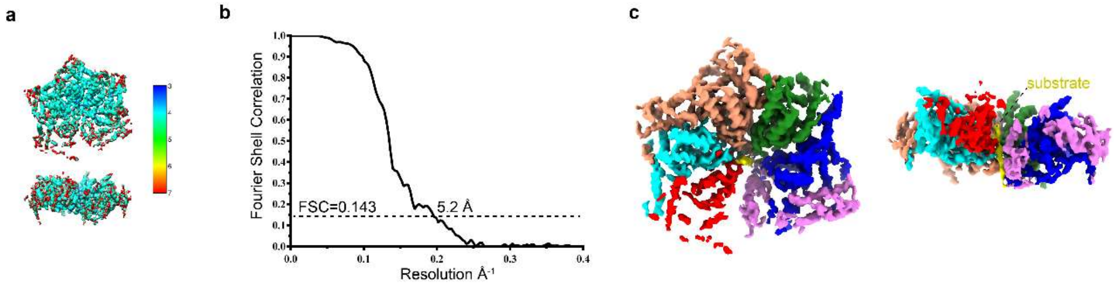

Rebuttal Figure 1. The density map of the NBD ring (double-hexamer). a-b, Local resolution estimation (a) and fourier shell correlation (FSC) curve (b) of the final map of the NBD ring in the $CLPB^{E425Q}$ double-hexamer. c, The density map of the NBD ring is displayed in surface representation, with individual NBD domains color coded. The substrate is colored yellow.

Why was AMP-PNP added in these experiments? The experiments shown in Fig. 2C appears critical in this context, but it remains unclear from the description in the legend whether or not ATPγS was present in all samples.

Non-hydrolysable nucleotide analogs, such as AMPPNP and ATPγS have been widely used in structural studies. Usually, with structures resolved at resolution of 3 Å or worse, they are not expected to induce significant structural differences to the enzymatic center. Both AMPPNP and ATPγS could still be hydrolyzed, but in much lower rates than ATP. Thus, they do have different kinetic properties, and as a result, functional experiments with these compounds could lead to contrasting results in certain cases (For example, see (Yao et al., 2022)). We initially chose AMPPNP for structural purpose is that it has a lower hydrolyzing rate. It should work the best to trap the enzymatic center in the pre-catalytic state.

Figure 2C (new Figure 2B) has been revised. The nucleotides are clearly indicated in the figure to avoid confusion.

Why was ATPγS rather than AMP-PNP added? Is the hexameric state induced upon substrate binding or only upon substrate binding in the presence of ATPγS? These experiments are central as they aim to establish a substrate induced change in the stoichiometry of CLPB complexes, a central and perhaps the most intriguing finding of the manuscript.

For the experiment of *in vitro* substrate (casein) binding, we have changed the nucleotide from AMPPNP to ATPγS. Based on a published study of Hsp104 (Gates et al., 2017), the affinity of the model substrate casein to Hsp104 was the highest in the presence of ATPγS (Kd: 20 nM for ATPγS vs 3.5 μM for AMPPNP) (Rebuttal Figure 2). For this reason, we have chosen to use ATPγS to maximize the binding of casein to the WT CLPB complex.

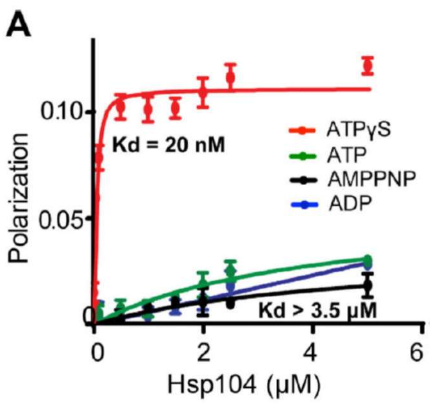

Rebuttal Figure 2. Binding of casein to Hsp104 in the presence of different nucleotides (Adapted from (Gates et al., 2017))

We fully understand the reviewer's concern on this issue. In the revision, we performed an additional experiment to test the change of the oligomeric state in the presence of ATPγS alone. As shown in Rebuttal Figure 3 (see also new Figure 2), the incubation of ATPγS alone only changed the oligomeric state for a very small fraction of particles (8%). Compared to the combination of ATPγS and casein, this percentage is neglectable. Therefore, we believe that our conclusion still holds true that the binding of substrate drives the transition from tetradecamers to dodecamers.

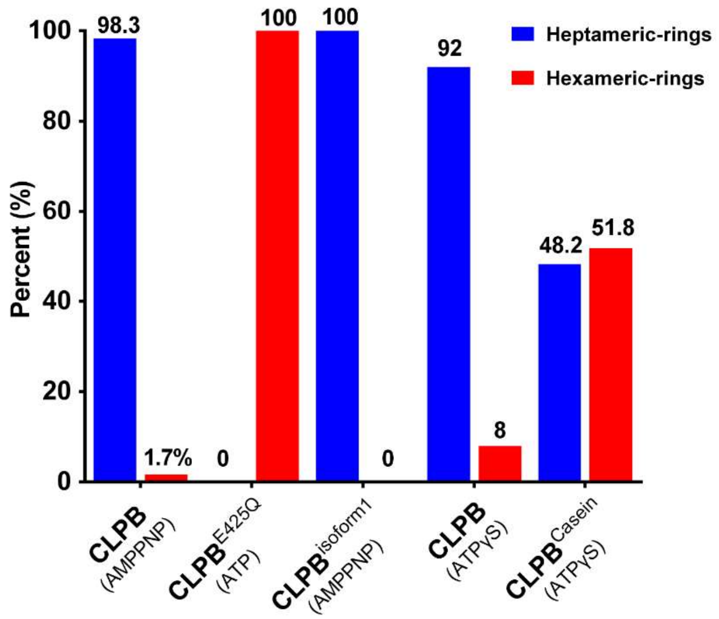

Rebuttal Figure 3. Oligomeric states of different CLPB complexes

2.The mass spectrometric analysis of CLPB interacting protein shown in Fig. 6 is not sufficiently documented and therefore difficult to assess. I could not find any description detailing the MS analysis of the data (number of biological replicates, statistical evaluation, etc).

In the revision, we have performed additional mass spectrometry analysis. Both the WT and mutant ANK domains were used for pull-down experiments. The final analyses were based on three biological replicates (starting from the expression plasmid transfection), and a stringent screening condition (P-value < 0.01 and Fold change > 4) was used. The procedures were included in the Method, and the MS data are provided in Supplemental Table S3.

If I understood correctly, MTS-ANK-HA was used as a control in this experiment. As the authors found that the deletion of the AA 201-232 in the ANK domain significantly reduces CLPB disaggregase activity and that this part of the protein might be involved in substrate recognition and transport of the substrate to AAA+ domain, such mutant (MTS-ANKΔ201-232Strep) would be great negative control in a search for the specific CLPB substrates.

This is a good suggestion. We have performed this experiment in the revision (see in new Figure 6). Compared with the mutant MTS-ANK$^{\Delta 201\text{-}232}$, 14 mitochondrial proteins in the OM, IMS, IM were more enriched in the WT-ANK sample (Rebuttal Figure 4), including a well-characterized CLPB-interacting protein HAX1 (Chen et al., 2019b; Fan et al., 2022). Please refer to the manuscript for more discussion (page 14, line 352-358)

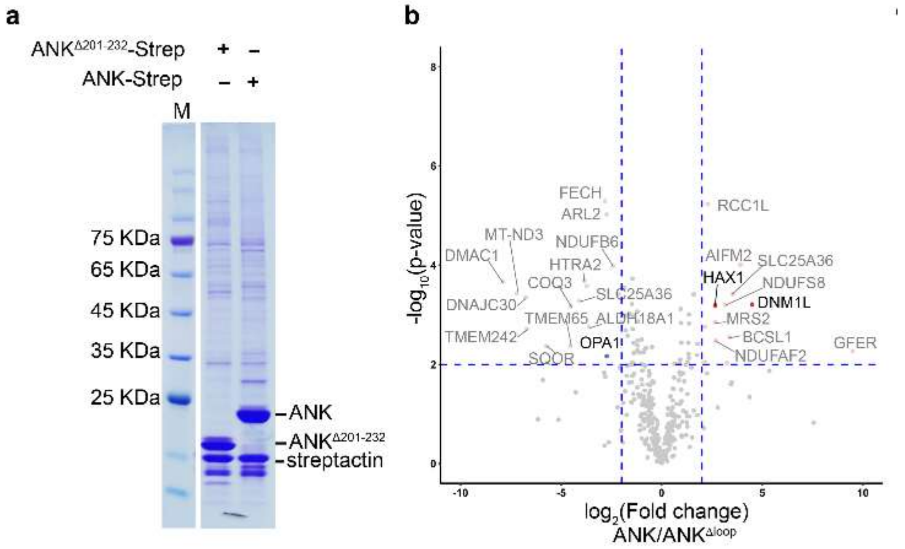

Rebuttal Figure 4. MS-based interactome analysis of ANK and ANK$^{\Delta 201\text{-}232}$. a HEK-293F cells expressing the MTS-ANK-Strep or MTS-ANK$^{\Delta 201\text{-}232}$-Strep constructs were subjected to anti-Strep immunoprecipitation. Precipitates were analyzed by SDS-PAGE, Coomassie blue staining and mass-spectrometry (MS). b, Volcano plot of the mitochondrial proteins co-precipitated with ANK-Strep based on a stringent screening condition (P-value < 0.01 and Fold change > 4).

Moreover, the experiments were seemingly performed in the presence of endogenous CLPB? Is endogenous CLPB found in the eluate? Experiments in CLPB knock-out cells would more informative.

The reviewer is correct that the MS experiments were done with HEK-293T cells of WT background. We could identify endogenous CLPB from the MS data. But the abundance of full-length CLPB is very low (Rebuttal figure 5), and its influence on the interactome analysis should be minimal.

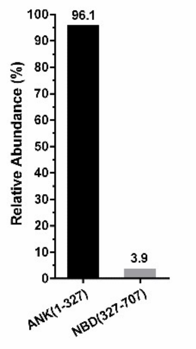

Rebuttal Figure 5. The relative abundance of ANK domain and NBD in the MS data.

3.The authors report that ANK motif deletion does not perturb ATPase activity (or even see higher ATPase activity of the NBD mutant). This result is in contrast to the published result by Cupo and Shorter that report lack of ATPase activity of the isolated NBD (consistent with a similar lack of activity of isolated NBD2 from yeast Hsp104 or bacterial ClpB). How do authors explain this discrepant result?

We have repeated our experiments (Rebuttal Figure 6) and the results are consistent with our previous observation: The NBD alone has higher ATPase activity than the full-length CLPB^{WT}. The ATP-binding site of AAA+ proteins is located between two neighboring protomers. Therefore, the ATPase activity of an NBD would critically dependent on its oligomeric state. In our study, as shown in Figure S9 and S10, CLPB-NBD alone forms helical oligomers, capable of binding to ATP and endogenous substrates. Thus, our structural and biochemical data are consistent. The relatively lower activity of the WT CLBP could be explained by the fact that the NBD ring of the tetradecamer is heptameric, not the hexamer form that is optimized for ATPase activity.

There is one major difference between ours and the published study (Cupo and Shorter, 2020). The pH value of the IMS was estimated to be 6.8 (Santo-Domingo and Demaurex, 2012). For this reason, we have chosen this condition for protein preparation and the ATPase assay. In contrast, in their work the buffer was pH 8.0 for the ATPase assay. The authors have stated in their response letter (published on the eLife website) that the NBDs in their study were not hexamers but a dimer-like species. Similarly, the reason that isolated NBD1 or NBD2 of ClpB has no or very low ATPase activity is because neither of them could form high-order oligomers (Beinker et al., 2005; Werbeck et al., 2011),

Therefore, we think that the discrepancy is well explained by the different buffer conditions in the two studies. In principle, our condition is more physiologically relevant.

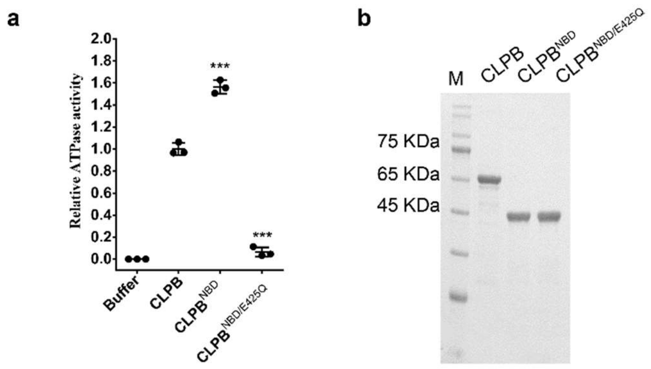

Rebuttal Figure 6. Double-checked the ATPase activity of NBD.

Reviewer #3:

Human CLPB (aka SKD3) is a protein disaggregase found in the mitochondrial intermembrane space, which promotes the solubilization of aggregated mitochondrial proteins (1, 2). CLPB consists of an ANK domain followed by a single AAA domain. It was previously shown that the ANK domain is essential to CLPB function (1) and missense mutations throughout the CLPB gene are associated with 3-methylglutaconic aciduria (3-MGA) and severe congenital neutropenia (SCN). Here, Wu et al. presents the cryo-EM structures of CLPB in the apo and casein-bound form. The reported oligomeric structures range from tetradecamers (heptamers), dodecamers (hexamers to nonamers. Although the work is technically sound, there are several inconsistencies and stark differences with published work that must be addressed.
The introduction is erroneous in many places, e.g. CLPB/SKD3 is NOT an Hsp78 homolog; the N-domain of yeast Hsp104 is dispensable for protein disaggregation and by extension substrate binding; the NBDs of Hsp100 do not elicit a power stroke.

We thank the reviewer for these corrections. The human gene of CLPB/SKD3 was also annotated as HSP78 in UniProt. We agree that the name of HSP78 is indeed very confusing. In the revision, we have removed the alternative name HSP78.

Yeast Hsp104 is capable of disassembling a variety of substrates, including disordered aggregates (Parsell et al., 1994), amyloids (DeSantis et al., 2012), and prions (Klaips et al., 2014; Shorter and Lindquist, 2004). The dependence of the disaggregase activity on the NTD varies largely among different substrates. We agree that our previous statement "the NTD is responsible for substrate binding and recognition" is not accurate. It has been revised.

By stating "the NBD1 and NBD2 bind and hydrolyze ATP to power substrate unfolding and translocation", we did not imply that Hsp104 elicits a power stoke. The sentence has been revised.

There is also substantial overlap with published work (e.g. Ref. 1) and the significance of the presented data are not always clear (e.g. Fig. EV3).

Some of these data were only functional validations of CLPB variant, since they were prepared from *E. coli* cells. In the revision, the figures have been largely reorganized (new Figure S6).

On balance, while the authors present a fine structure, the paper is rather confusing and highly speculative at times (nonamer structure), which precludes publication in its current form.
Major

1. The authors present cryo-EM structures of apo CLPB (heptamer/tetradecamer) and substrate-bound CLPB (hexamer/dodecamer) at medium resolution (~6-7 Å resolution). However, the authors did not further capitalize on this finding, and how CLPB goes from a heptamer to a hexamer is unclear.

We thank the reviewer for this suggestion. The first two structures we solved in our work is the WT CLPB and $CLPB^{E425Q}$. The double-heptameric form of the WT CLPB was very surprising. Therefore, we performed a few experiments to rule out the possible effects from exogenous *E. coli* expression and alternative splicing. The addition of a model substrate casein to WT CLPB proteins leads to an efficient conversion from the double-heptamer to the double-hexamer. In the revision, we further showed that the nucleotide ATPγS is not the determining factor for the change of its oligomeric state.

A highly plausible model is that the transition occurs on the double-heptamers without involving a complete disassembly of the complex. The binding of substrate to the pore-loops of the ATPase domains would bring close neighboring subunit (Figure 1) and result in a more compact conformation. The helical arrangement of subunits could be beneficial for the ejection of one subunit. In fact, in all published structures of AAA+ hexamers in substrate-engaged state, one subunit (the so-called seam subunit) is always very flexible (Gates and Martin, 2020), indicating that the intrinsic dynamics of CLPB could be sufficient to support the process. Given the head-to-head interaction between two opposite ANK domains, the dissociation of the two subunits, one from each heptamer, might be coupled in a certain way. In fact, there is no lateral interaction between adjacent ANK domains in a heptamer. Thus, structure-wise, this process is possible.

We have incorporated this model discussion in the revision. We fully agree that the model is speculative in nature and we plan to test this hypothesis in a future work.

2. The proposed heptamer (or tetradecamer) structure of apo CLPB is intriguing and contrasts published work showing that CLPB forms a hexamer both without substrate (3, 4) and with substrate (4). How can this be reconciled?

In the first work (Spaulding et al., 2022), the authors used sedimentation analysis and dynamic light scattering to characterize the oligomeric state of apo CLPB. What they have found is that CLPB forms a high-order oligomer other than the hexamer. Their data do not have enough resolution and precision to make a call between double-heptamer and double-hexamer (Rebuttal Figure 7). In fact, their conclusion was primarily based on the conventional notion

that AAA+ proteins form hexamers. It is also evident from their negative-staining EM results: one could not define the oligomeric state from those top-view average images. In our work, we used cryo-EM 2D classification to characterize the oligomeric state, and the difference between hexamer and heptamer is very clear.

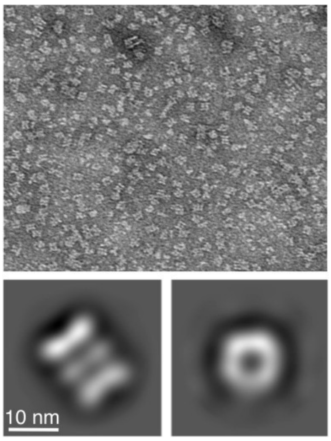

Rebuttal Figure 7. Negative-stain electron microscopy images of CLPB (Adapted from (Spaulding et al., 2022)).

In the second work (Cupo and Shorter, 2022), the authors in fact did not use cryo-EM to examine the sample of apo CLPB. Their structural analysis was based on particles of CLPB+ATPγS+casein. According to their results (Figure S1 in their preprint), the heptamer accounts for 19% of total particles. In our study, we discovered that the addition of casein (in the presence of ATPγS) could convert ~50% of complexes into double-hexamers, which was in a comparable range to their result, considering that the two studies used different concentrations of casein.

Therefore, we believe that the three studies had similar observations but the intepretations were different.

3. Perhaps even more perplexing is the structural discrepancy between apo CLPB (heptamer/tetradecamer) and CLPB-WB mutant (hexamer/dodecamer) co-purified with substrate. By co-purification, do the authors imply an E. coli protein that co-purified with CLPBWB? What is the identity of the bound protein and why is the bound polypeptide not shown in Fig. 1? As stated, the logic of a co-purified substrate-bound CLPB-WB is a stretch.

The peptide seen in the central channel of the mutant CLPB complexes should be a mixture of non-specific proteins of *E. coli* that happened to be captured by exogenously expressed CLPB complexes in *E. coli* cells. As shown in structures of the CLPB-NBD complexes (Figure 6), the main chain of the bound peptide is well resolved, but the side-chains are only resolved up to the β-carbon atoms, indicating a heterogeneity for bound substrates.

As we have shown in Figure S4, the density map of the NBD ring in the mutant CLPB complex could be improved to 5.2-Å resolution, which is sufficient to identify the bound substrate.

Figure 1 was prepared with the global density map to highlight the architectural difference between WT and mutant CLPB complexes (please also refer to our response to related questions from Reviewer #2). As suggested, we have labeled the substrate in the revised Figure 1.

The E-to-Q Walker-B mutation was commonly used for structural purposes. This mutation largely abolishes ATP hydrolysis without compromising nucleotide binding of ATP/ADP. In recent years, cryo-EM studies on different AAA+ proteins have repeatedly found that the introduction of this mutation resulted in a stable binding of co-purified substrate, including ClpX (Fei et al., 2020), ClpB (Yu et al., 2018), Vps4 (Monroe et al., 2017), AFG32L (Puchades et al., 2019), YME1 (Puchades et al., 2019) and Rix7 (Kocaman et al., 2022; Lo et al., 2019). It is now becoming a common practice in structural studies for capturing substrate-bound state. By doing so, we only meant to use it as a structural model to illustrate the interaction between the substrate and the pore-loops of CLPB.

4. Heptamers, incl. tetradecamers, have been reported for several AAA+ motors including Clp/Hsp100 (c.f. also extensive literature on p97), but are largely dismissed as structural artifacts. Cupo et al. (4) previously showed that heptamers are a minor species contrasting the data presented in Fig. 2C. To test whether a CLPB heptamer/tetradecamer is the predominant species, the authors need to independently verify their heptamer structure by site-specific cross-linking and gel analysis, or mass photometry.

We fully understand the reviewer's concern. As we have pointed out in our response to question #2, Cupo and co-workers did not explicitly examine the oligomeric states of apo CLPB. Based on their data, in the presence of the model substrate casein, 19% of particles are still in the form of double-heptamer. In our work, two indepentents results have supported that the double-heptamer is the predominant species of apo CLPB. The first is gel filtration (Supplementary Fig. 1), in which double-hexamers ($CLPB^{E425Q}$) were indeed eluated slighly later than double-heptamers. The second is cryo-EM: we showed that apo CLPB, apo CLPB (purified from mamalian cells), apo $CLPB^{isoform1}$ all primarily form double-heptamers (Figure 2; Supplementary Fig. 2). During image processing, we have treated the particles unbiasedly during 2D and 3D classification. Therefore, we are confident that the predomiant species of apo CLPB is a double-heptamer.

It remains to be examined whether such a double-heptamer exist in the cell. We hope the reviewer to understand that we feel unnecessary to further verfiy this oligomeirc state *in vitro*. We wish to explore the *in vivo* relevence of this oligomeric state in a future study.

5. In addition to the widely observed hexamer/heptamer structures, the authors also observed a highly speculative nonamer structure at 3.7-Å resolution. Although the authors state that the nonamer structure is non-physiological, it is important to validate any structural insight derived from an artifactual structure. To test whether the nonamer structure has any functional relevance, independent biochemical data using CLPB mutants must be presented. If not, the nonamer structure is confusing and does not add to the conclusion.

The first two structures we have determined are apo CLPB and CLPB^{E425Q}. Unfortunately, neither of them was solved at a sufficient resolution for atomic modelling. Therefore, we further determined the cryo-EM structures of CLPB-NBD and the crystal structure of the ANK domain. Therefore, the purpose of these domain structures is to provide high-resolution templates for structural interpretation of double-heptamer and double hexamer. The helical structures of CLPB-NBD also provide a structural basis for understanding the interaction between peptide substrate and pore-loops of CLPB. What we have observed in the helical structures are consistent with the current knowledge of AAA+ hexamers. Therefore, these structures, although non-physiological, indicate that CLPB shares common structural features with classical AAA+ proteins.

We have to mentioned that the 3.7-Å resolution is an average number for the overall complex. As shown in Supplemental Figure S10, the core of complex is better than 3.0 Å. In this sense, the atomic model from this complex is important and could not be replaced by AlphaFold2 predicted models.

6. Hsp100 motors translocate substrate in a unidirectional manner and bind substrates via their N-domains (here: ANK domain). In the present structure, CLPB adopts a head-to-head oligomer held together by the ANK domains, which precludes a substrate interaction via the CLPB N-domain. Since the ANK domains mediate multiple interactions with neighboring subunits both within one oligomer and across oligomers, how do they engage with substrate (not to mention large aggregates)?

As shown in Figure 1, the ANK domain only mediates head-to-head dimerization. There is no contact between adjacent ANK domains within a heptamer or hexamer. In addition, the inner chamber of CLPB is freely accessible from the large open space between subunits. Therefore, we think that the ANK ring of CLPB complex is not a physical barrier for substrate interaction. In the case of a model substrate casein, the flexible terminal sequence (S337-Q391, 55 amino acids in the C terminus) could easily diffuse into the chamber.

In the case of misfolded aggregates, the situation is more complex. Our updated MS data support a direct role of the ANK domain in substrate engagement. This is consistent with a general role of ankyrin repeats in the protein-protein interactions (Parra et al., 2015). Of note, a very recent NMR study on Hsp104 has identified a substrate-binding groove in the NTD to recognize exposed hydrophobic stretches in substrates of Hsp104 (Harari et al., 2022)). The ANK domain may work in a similar fashion to bind to exposed hydrophobic sequences of the substrate. It has to be noted that the ANK domain indeed contain two hydrophobic surface patches (Figure 3B).

7. The mass spectrometry data presented in Fig 6C is interesting and largely consistent with published work (1, 5). However, according to MitoCarta 3.0, HAX-1 is not an IMS protein and may not even be a mitochondrial protein at all. A discussion is required.

The suggestion is well taken. We have added relevant discussion in the revision. HAX1 was

not annotated as a mitochondrial protein in MitoCarta 3.0 (Rath et al., 2021). Based on published studies, HAX1 is a protein of multiple cellular location. Its mitochondrial location was in fact confirmed by many independent studies. HAX1 was first identified as a mitochondrial protein in 1997 (Suzuki et al., 1997). Suzuki and co-workers have showed that 1) HAX1 co-immunoprecipitated with a mitochondrial protein HS1; 2) HAX1, HS1 and Bcl-X (as an outer membrane marker) co-localized to mitochondria based on fluorescent microscopy. Other studies have also demonstrated a mitochondrial location for HAX1 using different mitochondrial markers (for examples, (Vafiadaki et al., 2009; Vafiadaki et al., 2007)). Most recently, a study employed proteinase K assay on isolated mitochondria and showed that the degradation of HAX1 (CLPB as well) requires the disruption of the outer membrane (Fan et al., 2022). Therefore, we think that it could be a mis-annotation of MitoCarta 3.0.

8. There are several erroneous statements in the introduction that need to be corrected. For instance, CLPB is a single-ring oligomer while ClpB (as well as Hsp78 and Hsp104) forms a two-ring hexamer. In essence human CLPB and microbial ClpB/Hsp104/Hsp78 belong to different Hsp100 classes. Hence, the term "human CLPB" is a misnomer since the protein is both structurally and functionally unrelated to bacterial ClpB. A re-review of the revised manuscript is imperative to ensure that statements made are factually correct.

Thank you. We have revised the text to avoid confusions.

Minor

1.The Li et al., 2006 reference to support "…first protein…to contain both ANK domain and AAA+ activity" seems misplaced.

Revised.

2.A Glu to Gln mutation of the Walker B does not necessarily abrogate ATP hydrolysis since a Gln is fully compatible with a nucleophilic attack. Independent biochemically verification is required.

Suggestion is well taken. We also tested the mutant of $CLPB^{E425A}$. There is no difference in their effects on the ATPase activity of CLPB. Both of them are inactive in hydrolyzing ATP.

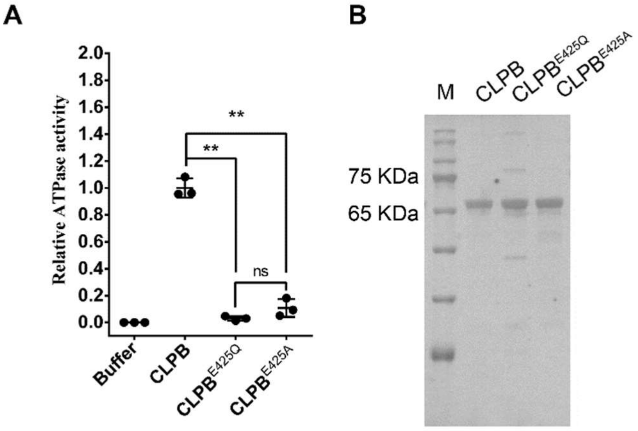

Rebuttal Figure 8. The ATPase activity of CLPB^{E425Q} and CLPB^{E425A}.

3.Fig. EV1: It is widely appreciated that AMP-PNP introduces a structural artifact in Hsp100 proteins and is incompatible with the true "ATP-activated" state required for substrate binding. The Glu to Gln mutation in the Walker B motif is more physiological but does not necessarily abrogate ATP hydrolysis.

We understand the reviwer's concern. In the revision, we have performed a validation experiment on the oligomeric state of the apo CLPB. ATPγS was incubated with WT CLPB and cryo-EM 2D classification was used to estimate the percentage of hexamers in all top-view particles. Under this condition, a majority (92%) of WT CLPB is still in the form of double-hepatmer.

We also tested the ATPase activity of mutant CLPB^{E425A}. Both E425Q and E425A mutations are defective in hydrolyzing ATP (Rebuttal Figure 8). Thus, it indicates that for CLPB Walker-B E425Q is an effective mutant.

4. Data presented in Fig. EV3 are largely identical to published work (1).

This figure serves as an introduction to the construct design and a validation of purified proteins. The data has been reorganized in the revision.

5. Please use line and page numbers in any revision.

We added the line and page numbers in the revision.

References

Cupo RR, Shorter J. Skd3 (human CLPB) is a potent mitochondrial protein disaggregase that is inactivated by 3-methylglutaconic aciduria-linked mutations. eLife. 2020;9:e55279.

Mróz D, Wyszkowski H, Szablewski T, Zawieracz K, Dutkiewicz R, Bury K, et al. CLPB (caseinolytic peptidase B homolog), the first mitochondrial protein refoldase associated with human disease. Biochim Biophys Acta. 2020;1864(4):129512.

Spaulding Z, Thevarajan I, Schrag LG, Zubcevic L, Zolkiewska A, Zolkiewski M. Human mitochondrial AAA+ ATPase SKD3/CLPB assembles into nucleotide-stabilized dodecamers. Biochem Biophys Res Commun. 2022;602:21-6.

Cupo RR, Rizo AN, Braun GA, Tse E, Chuang E, Southworth DR, et al. Unique structural features govern the activity of a human mitochondrial AAA+ disaggregase, Skd3. bioRxiv. 2022:2022.02.17.480866.

Chen X, Glytsou C, Zhou H, Narang S, Reyna DE, Lopez A, et al. Targeting mitochondrial structure sensitizes acute myeloid leukemia to venetoclax treatment. Cancer Discov. 2019;9(7):890-909.

Reviewer #4 (Christopher Hill, signs review):

Structural and biochemical studies are reported of CLPB, a mitochondrial AAA disaggregase that is linked to human disease and is unusual among AAA unfoldases in having an N-terminal ANK domain.
Curiously, it is found that the ANK domain mediates face-to-face stacking of AAA ring structures, such that the active state of CLPB is proposed to be a dodecamer of two hexameric rings facing each other rather than the single hexameric ring envisioned for other AAA unfoldases. In the absence of bound substrate, CLPB is seen to form a tetradecamer in which two heptameric rings face each other in a configuration that is envisioned to be inactive.
Structures were also determined of the NBD lacking the ANK domain, and seen to bind substrate in hexameric, heptameric, and nonameric forms. Although the nonamer is envisioned to be not relevant physiologically, it gave the best structure and view of substrate interactions at 3.7Å resolution.
I do not have any technical concerns.

There are numerous instances where the use of English should be improved.

Thank you. We have carefully revised the manuscript to avoid grammar mistakes and to improve the language.

I am doubtful that the precision implied by the description of hydrogen bonding interactions is justified by the resolution of the reconstructions.

As shown in the Supplemental Figure S10, the core region of the nonamer in the density map could reach as good as 3-Å resolution. For resolution estimation, we used a loose mask to calculate the FSC curve. In fact, PL-Is are in the best resolved region of the nonamer, due to their interactions with the peptide substrate (Fig. 5, Rebuttal Figure 9).

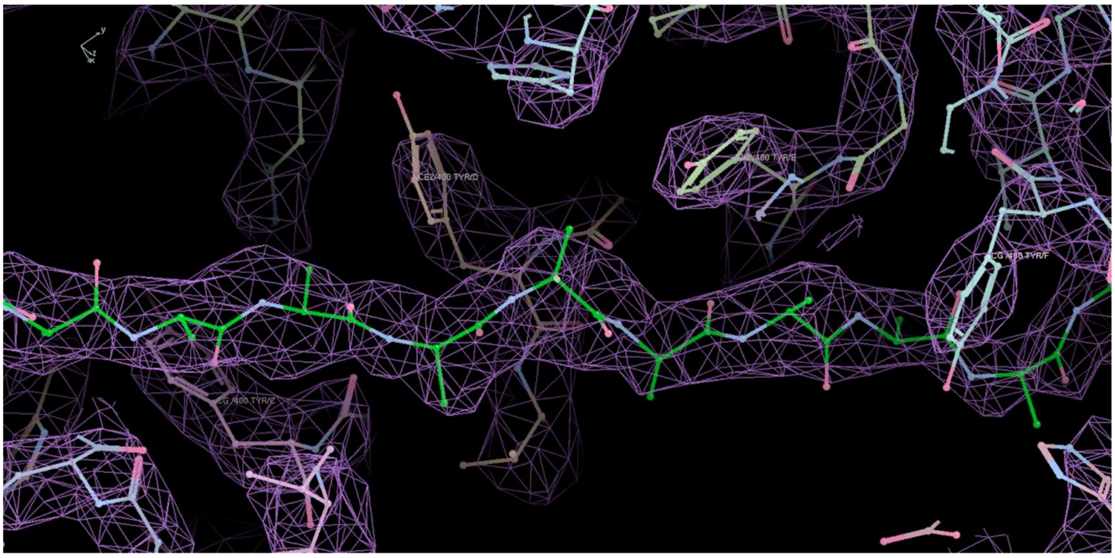

Rebuttal Figure 9. The density map of NBD nonameric complex.

I am confused about the axial dimensions in Figure 4. Given that the substrate is modeled in an extended/beta conformation with two residues and 60° rotation per subunit, the axial displacement per subunit (24/6, 29/6, 32/6; 4-5.3Å/subunit) all seem too short. Given that the axial displacement is almost parallel to the substrate strand, I would expect axial displacements in the range of 6-7Å per subunit.

The three oligomer of NBD are not in perfect helical symmetry. The axial rise between subunits is not constant in each oligomer and varies in the hexamer and heptamer largely. In the density map of the hexamer, the P1 subunit is flexible (Supplemental Figure S10, local resolution map) and its PL-I is not attached to the substrate. The P1 in the hexamer is the so-called "seam" subunit of AAA+ hexamers. In the map of the heptamer, both the P1 and P7 are relatively unstable (Figure 4B). During the 3D classification, we noticed that the core regions (P3, P4 and P5) of the three oligomers could be perfectly aligned with each other, indicating that they share same axial rise for these three subunits. But in the hexamer, the axial distances between P6 and P5, and between P1 and P2 are both very small.

In the revision, we have remeasured the distance using the central peptide as a reference, and rounded the numbers due to precision of the measurement. Please note that the lines between two Y400 Cα atoms of P1 and P6 are not vertical (one subunit offset). In the nonamer, a stable binding between the PL-I and the substrate is observed for all subunits, the axial distance between P1 and P6 should be approximately the backbone length of 5×2=10 amino acid residues (roughly 35 Å).

The authors propose that two hexamers may act on substrate in a coordinated manner within the dodecameric assembly. It is not clear to me, however, that an alternative and more conventional model has been disproven - specifically, that the ANK domains function to localize CLPB to substrate, perhaps by binding extended portions of protein aggregates, while single hexameric rings translocate/unfold the substrate. The authors may want to modify the discussion to acknowledge this possibility.

We thank the reviewer for this good suggestion. Initially, we hope to find an ANK mutation to separate its two functions (substrate binding and structural organization). So far, the two tested mutations (R178E and R227E at the ANK dimer interface) could not serve this purpose. They did not disrupt the high-order assembly, but on the contrary impaired the disaggregase activity.

In the revision, we first discussed the conventional model and presented the current model as an interesting alternative model (Page 14).

References

Beinker, P., Schlee, S., Auvula, R., and Reinstein, J. (2005). Biochemical coupling of the two nucleotide binding domains of ClpB: covalent linkage is not a prerequisite for chaperone activity. J Biol Chem *280*, 37965-37973.

Chen, X., Glytsou, C., Zhou, H., Narang, S., Reyna, D.E., Lopez, A., Sakellaropoulos, T., Gong, Y., Kloetgen, A., Yap, Y.S.*, et al.* (2019). Targeting Mitochondrial Structure Sensitizes Acute Myeloid Leukemia to Venetoclax Treatment. Cancer Discov *9*, 890-909.

Cupo, R.R., and Shorter, J. (2020). Skd3 (human ClpB) is a potent mitochondrial protein disaggregase that is inactivated by 3-methylglutaconic aciduria-linked mutations. Elife *9*.

DeSantis, M.E., Leung, E.H., Sweeny, E.A., Jackrel, M.E., Cushman-Nick, M., Neuhaus-Follini, A., Vashist, S., Sochor, M.A., Knight, M.N., and Shorter, J. (2012). Operational plasticity enables hsp104 to disaggregate diverse amyloid and nonamyloid clients. Cell *151*, 778-793.

Fan, Y., Murgia, M., Linder, M.I., Mizoguchi, Y., Wang, C., Lyszkiewicz, M., Zietara, N., Liu, Y., Frenz, S., Sciuccati, G.*, et al.* (2022). HAX1-dependent control of mitochondrial proteostasis governs neutrophil granulocyte differentiation. J Clin Invest *132*.

Fei, X., Bell, T.A., Jenni, S., Stinson, B.M., Baker, T.A., Harrison, S.C., and Sauer, R.T. (2020). Structures of the ATP-fueled ClpXP proteolytic machine bound to protein substrate. Elife *9*.

Gates, S.N., and Martin, A. (2020). Stairway to translocation: AAA+ motor structures reveal the mechanisms of ATP-dependent substrate translocation. Protein Sci *29*, 407-419.

Gates, S.N., Yokom, A.L., Lin, J., Jackrel, M.E., Rizo, A.N., Kendsersky, N.M., Buell, C.E., Sweeny, E.A., Mack, K.L., Chuang, E.*, et al.* (2017). Ratchet-like polypeptide translocation mechanism of the AAA+ disaggregase Hsp104. Science *357*, 273-279.

Harari, A., Zoltsman, G., Levin, T., and Rosenzweig, R. (2022). Hsp104 N-terminal domain interaction with substrates plays a regulatory role in protein disaggregation. FEBS J.

Klaips, C.L., Hochstrasser, M.L., Langlois, C.R., and Serio, T.R. (2014). Spatial quality control bypasses cell-based limitations on proteostasis to promote prion curing. Elife *3*.

Kocaman, S., Lo, Y.-H., Krahn, J.M., Sobhany, M., Dandey, V.P., Petrovich, M.L., Etigunta, S.K., Williams, J.G., Deterding, L.J., Borgnia, M.J.*, et al.* (2022). Communication network within the essential AAA-ATPase Rix7 drives ribosome assembly. PNAS Nexus.

Lo, Y.H., Sobhany, M., Hsu, A.L., Ford, B.L., Krahn, J.M., Borgnia, M.J., and Stanley, R.E. (2019). Cryo-EM structure of the essential ribosome assembly AAA-ATPase Rix7. Nat Commun *10*, 513.

Monroe, N., Han, H., Shen, P.S., Sundquist, W.I., and Hill, C.P. (2017). Structural basis of protein translocation by the Vps4-Vta1 AAA ATPase. Elife *6*.

Parra, R.G., Espada, R., Verstraete, N., and Ferreiro, D.U. (2015). Structural and Energetic Characterization of the Ankyrin Repeat Protein Family. PLoS Comput Biol *11*, e1004659.

Parsell, D.A., Kowal, A.S., Singer, M.A., and Lindquist, S. (1994). Protein disaggregation mediated by heat-shock protein Hsp104. Nature *372*, 475-478.

Puchades, C., Ding, B., Song, A., Wiseman, R.L., Lander, G.C., and Glynn, S.E. (2019). Unique Structural Features of the Mitochondrial AAA+ Protease AFG3L2 Reveal the Molecular Basis for Activity in Health and Disease. Mol Cell *75*, 1073-1085 e1076.

Puchades, C., Sandate, C.R., and Lander, G.C. (2020). The molecular principles governing the activity and functional diversity of AAA+ proteins. Nat Rev Mol Cell Biol *21*, 43-58.

Rath, S., Sharma, R., Gupta, R., Ast, T., Chan, C., Durham, T.J., Goodman, R.P., Grabarek, Z., Haas,

M.E., Hung, W.H.W., *et al.* (2021). MitoCarta3.0: an updated mitochondrial proteome now with sub-organelle localization and pathway annotations. Nucleic Acids Res *49*, D1541-D1547.
Santo-Domingo, J., and Demaurex, N. (2012). Perspectives on: SGP symposium on mitochondrial physiology and medicine: the renaissance of mitochondrial pH. J Gen Physiol *139*, 415-423.
Shorter, J., and Lindquist, S. (2004). Hsp104 catalyzes formation and elimination of self-replicating Sup35 prion conformers. Science *304*, 1793-1797.
Spaulding, Z., Thevarajan, I., Schrag, L.G., Zubcevic, L., Zolkiewska, A., and Zolkiewski, M. (2022). Human mitochondrial AAA+ ATPase SKD3/CLPB assembles into nucleotide-stabilized dodecamers. Biochem Biophys Res Commun *602*, 21-26.
Suzuki, Y., Demoliere, C., Kitamura, D., Takeshita, H., Deuschle, U., and Watanabe, T. (1997). HAX-1, a novel intracellular protein, localized on mitochondria, directly associates with HS1, a substrate of Src family tyrosine kinases. J Immunol *158*, 2736-2744.
Vafiadaki, E., Arvanitis, D.A., Pagakis, S.N., Papalouka, V., Sanoudou, D., Kontrogianni-Konstantopoulos, A., and Kranias, E.G. (2009). The anti-apoptotic protein HAX-1 interacts with SERCA2 and regulates its protein levels to promote cell survival. Mol Biol Cell *20*, 306-318.
Vafiadaki, E., Sanoudou, D., Arvanitis, D.A., Catino, D.H., Kranias, E.G., and Kontrogianni-Konstantopoulos, A. (2007). Phospholamban interacts with HAX-1, a mitochondrial protein with anti-apoptotic function. J Mol Biol *367*, 65-79.
Werbeck, N.D., Zeymer, C., Kellner, J.N., and Reinstein, J. (2011). Coupling of oligomerization and nucleotide binding in the AAA+ chaperone ClpB. Biochemistry *50*, 899-909.
Wortmann, S.B., Zietkiewicz, S., Kousi, M., Szklarczyk, R., Haack, T.B., Gersting, S.W., Muntau, A.C., Rakovic, A., Renkema, G.H., Rodenburg, R.J., *et al.* (2015). CLPB mutations cause 3-methylglutaconic aciduria, progressive brain atrophy, intellectual disability, congenital neutropenia, cataracts, movement disorder. Am J Hum Genet *96*, 245-257.
Yao, N.Y., Zhang, D., Yurieva, O., and O'Donnell, M.E. (2022). CMG helicase can use ATP gamma S to unwind DNA Implications for the rate-limiting step in the reaction mechanism. P Natl Acad Sci USA *119*.
Yu, H., Lupoli, T.J., Kovach, A., Meng, X., Zhao, G., Nathan, C.F., and Li, H. (2018). ATP hydrolysis-coupled peptide translocation mechanism of Mycobacterium tuberculosis ClpB. Proc Natl Acad Sci U S A *115*, E9560-E9569.
