# Supplementary material for: Comprehensive structural characterization of the human AAA+ disaggregase CLPB in the apo- and substrate-bound states reveals a unique mode of action driven by oligomerization
Source: PLoS Biol. 2023 Feb 6;21(2):e3001987. doi: 10.1371/journal.pbio.3001987 (PMC9934407; doi:10.1371/journal.pbio.3001987)
Supplement: Attachment — Submitted filename: Rebuttal-R3-NG.docx [file pbio.3001987.s025.docx]

**Response to Editor:**

\# I am writing to let you know that after our discussions, we understand that you disagree with Reviewer #3 and you are welcome to address the issues in the manuscript text without providing further experiments. We ask that you please provide a full and scholarly rebuttal that explains in detail why any additional experiments have not been performed. We will then evaluate the rebuttal and discuss the revised manuscript with the Academic Editor.

We appreciate editors’ understanding of our opinions on the comments from Reviewer #3. In this new revision, we have provided a full rebuttal and revised our manuscript.

#In addition, we also ask that you please discuss and compare your results with the recent Cell Reports paper given that it has now been published.

The suggestion is well taken. We have fully integrated the results from Cupo et al. (2022) into our manuscript.

**Reviewer #1 (Masafumi Yohda, signs review):**

The authors answered to all queries from the reviewers and revised the manuscript according to the suggestions. Thus, I recommend this manuscript for publication.

RE: We thank Reviewer #1 for his expert opinion and recommendation to publish our manuscript.

**Reviewer #2 (Thomas Langer, signs review):**

The authors have carefully considered points of criticisms on their original manuscript and significantly improved their manuscript. The newly added MS data on interaction partners of ANK domains and mutants thereof are convincing and strengthen the conclusion of the authors. This is an interesting manuscript providing new insight as to how CLPB handles its substrate, which will be of broad interest.

RE: We thank Reviewer #2 for his expert opinion and appreciation of our interactome data.

**Reviewer #3:**

Wu et al reports the cryoEM structures of apo CLPB (double-heptamer) and ATP-bound CLPB with a bacterial substrate mimic (double-hexamer) at medium resolution. Based on these findings, the authors propose that the double-heptamer may represent a resting state, which is a new finding. The 3D structure of the double-hexamer with casein was recently published by Cupo et al Cell Rep, 2022. Although, both structures consist of a double-hexamer, Cupo et al favors that the active form is a hexamer consistent with other single-ring Hsp100 unfoldases, contrasting the present work.

RE: Cupo et al. did not state that the active form of CLPB is a hexamer exclusively. Instead, they proposed that the active, substrate-bound form of Skd3 likely exists in a dynamic equilibrium between hexamers and dodecamers, and the dodecamer formation may enhance CLPB disaggregase activity (Cupo et al., 2022).

The following sentences are directly quoted from their paper. (1) “We suggest that the active, substrate-bound form of _PARL_Skd3 likely exists in a dynamic equilibrium between hexamers and dodecamers”; (2) “By contrast, our findings suggest that dodecamer formation may enhance _PARL_Skd3 disaggregase activity. _PARL_Skd3 hexamers and dodecamers are in dynamic equilibrium, but the dodecamer predominates upon polypeptide binding. The head-to-head ANK contacts could concentrate _PAR_LSkd3 disaggregases on the aggregate surface and enable stronger pulling forces by maximizing the number of hexamers.”

As we have stated in the discussion (the third paragraph of Discussion) (Page 13), we also discussed two possible models. One is that the hexamers work independently; the other is that the two hexamers work in a synergistic manner.

Therefore, we do not think there is essential difference between ours and their structural data, or between ours and their interpretations.

A shortcoming of the present study is the lack of new mechanistic insight since both the 3D structure and MS data have been published by others.

RE: The two structural studies by us and by Cupo et al. are independently done and theirs was only published during the second round of review of our manuscript.

In terms of the MS data, as far as we know, there is no systematic interactome analysis on binding partners of CLPB. From published data, only three studies related to the function of CLPB have employed MS-based analysis.

Chen *et al.* found that CLPB is upregulated in human acute myeloid leukemia (AML), and with a genome-wide screening identified that CLPB mediates Venetoclax (a BCL2 inhibitor) resistance in AML blasts (Chen et al., 2019). In their work, they performed immunoprecipitation (IP) of the endogenous human CLPB, followed by simple MS-based identification (no replicate). They identified mitochondrial proteins as CLPB interactors based on their presence in the CLPB pulldown and absence in the IgG control sample. HAX1 and OPA1 were among the top list of enriched proteins.

Cupo and Shorter showed that CLPB is a potent mitochondrial protein disaggregase. They empolyed MS (three replicates) to analyze the mitochondrial protein solubility in WT and CLPB-knockout cells. They found that many mitochondrial proteins exhibit decreased protein solubility in the knockout cells, including HAX1 and OPA1 (Cupo and Shorter, 2020).

Fan et al. reported that CLPB and HAX1 control mitochondrial proteostasis to regulate the differentiation and function of neutrophil granulocytes (Fan et al., 2022). In their work, they identified CLPB as one of the most prominent binding partners of HAX1 by transfecting FLAG-tagged HAX1 into HEK293T cells and performing IP experiments and subsequent MS identification (no replicate).

As summarized above, among the three studies, only one of them is related to direct binding partners of CLPB (Chen et al., 2019). But the study was not carried out in a quantitative manner, and it offered no information on potential direct binding proteins of the ANK domain.

In contrast, we used the ANK domain (both the WT and mutants) for pull-down and carried out quantitative mass spectrometry to systematically analyze the interactome of CLPB-ANK. Reviewer #2 Tomas Langer, who is a well-known expert in mitochondrial biology, has indicated that “The newly added MS data on interaction partners of ANK domains and mutants thereof are convincing and strengthen the conclusion of the authors”.

It is not so much that different oligomer states are observed without and with polypeptide, but how CLPB goes from a double-heptamer to a double-hexamer, which is of interest to the field. The authors propose that two CLPB subunits are "ejected" upon substrate binding, which seems implausible considering the double-ring structure. Furthermore, flexibility at the seam is very different from removal of CLPB subunits that are held within the ring complex and across rings via the ANK domain.

RE: As shown in Figure 2, under *in vitro* conditions, we discovered that the addition of substrates induces the transition from double-heptamers to double-hexamers. In the presence of ATPγS and casein, around 50% of double-heptamers could be converted into double-hexamers. This observation was in fact confirmed independently by Cupo et al. (2022). In their dataset of CLPB+ATPγS+casein, they also found a population of double-heptamers (Rebuttal Figure 1). The ratio of double-heptamers during 3D classification in their study is 19% (Class 3 in Rebuttal Figure 1). Considering that the two studies used different casein proteins in different concentrations, the two numbers (19% vs 48%) are totally comparable. And they had a similar interpretation: “… in the presence of FITC-casein, _PARL_Skd3 shifted toward dodecamers in all nucleotide conditions. Thus, substrate binding by _PARL_Skd3 promotes dodecamerization.” (Page 2 of their paper)

Rebuttal Figure 1. 2D and 3D Classification results of CLPB-Casein by Cupo et al. [Adapted from (Cupo et al., 2022)]

Therefore, we believe that the substrate induced change on oligomeric state is a feature of CLPB. Regarding the mechanism of this switch in oligomeric state, one has to employ single-molecule biophysical techniques (for example, FRET). But these experiments would require a substantial amount of work and could take at least 6 months or even years.

While the presented cryoEM structures are solid, they appear to be non-physiological, of medium resolution, and more geared towards the specialist.

As we have stated in the previous rebuttal letter and also in the manuscript, the structures of different forms in our study serve different purposes.

(1) The double-heptamer and double-hexamer are full-length structures. As discussed in our manuscript, several factors contribute to the inter-hexamer and intra-hexamer flexibility (Page 5), and therefore they were not resolved at high-resolution for the global density map. This is in fact also true for the study by Cupo et al. (2022), and the complete double-hexamer in their study was not resolved at high resolution either (For their map appearances, see Figure 1, Figure 3, Figure S1F, Figure S2E, Figure S3L in their paper).

(2) The structures of NBD^E425Q^ (substrate bound) is to provide high-resolution templates for structural interpretation of double-heptamer and double hexamer. In the high-resolution structures of the NBD^E425Q^, the PL-Is of the protomers form spiral staircases around the substrate, similar to other AAA+ unfoldases/disaggregases.

(3) To facilitate the analysis of the ANK interface, we also solved the crystal structure of the ANK domain.

* Fig 2B and Rebuttal Fig 3 suggest that only CLPB(E425Q) with ATP forms a double-hexamer. ATP analogs give rise primarily to heptamers with casein driving the formation of hexamers. The latter is consistent with the published work by Cupo et al., 2022.; who only observed double-hexamers.

RE: Cupo et al. also observed the double-heptamers (Rebuttal Figure 1) (Cupo et al., 2022). The description in their paper, “Following 3D classification with four classes, …… and a heptameric form containing two rings and an empty central channel (class3)”.

No two 3D reconstructions are alike and whatever the correct answer might be, according to the present study, it would seem that the CLPB structures with AMP-PNP and ATPγS represent off-pathway states.

RE: Non-hydrolysable nucleotide analogs, such as AMPPNP and ATPγS have been widely used in structural studies. Cupo et al. also used ATPγS in the study for structural determination of various CLPB complexes. In our study, the structure of the apo double-heptamer was obtained in the presence of AMPPNP, and the structures of double-hexamer and NBD in the presence ATP. The analog of ATPγS was used to analyze the oligomeric state change.

We think that it is not fair to simply state that these structures are off-pathway states. In structural studies, the analog-arrested structures represent stable *in vitro* 3D conformations of a certain sample, and they should, if not completely, at least reflect some aspects of the 3D structures *in vivo*.

* Heptamer structures were observed with different AAA+ proteins, often at low resolution, and frequently by cryoEM, and are largely dismissed as an in vitro artifact. The fact that heptamers and other oligomeric species are being reported in the literature (lines 292-294) does not make them physiological. Of note, a bacterial homolog of Bcs1 was recently shown to be a hexamer.

RE: In the manuscript, we did not make a claim that the double-heptamer is absolutely physiological. In the Discussion, we only discussed the possibility of a resting state for CLPB as a double-heptamer (Page 12). The following paragraph is directly copied from our manuscript.

“It is not clear whether the double-heptamer is a physiological state of CLPB *in vivo*. But several lines of evidence appear to suggest it is not a not a pure in vitro artifact. First, the heptamer form is the predominant species of apo CLPB (Fig 2), indicating that it is an intrinsic property of the CLPB ATPase module. Although AAA+ proteins are generally considered to be hexamers, an increasing number of AAA+ proteins were recently discovered to take heptamer as a primary oligomeric state (Hoffmann et al., 2022; Kater et al., 2020; Kim et al., 2020; Tang et al., 2020), including a mitochondrial IM-bound AAA+ protein Bcs1 (Kater et al., 2020; Tang et al., 2020). Second, the substrate-induced change on the oligomeric state has also been observed for other AAA+ members, such as DNA helicase AAV2 Rep68 (Santosh et al., 2020), RuvB (Miyata et al., 2000), and archaeal MCM (Yu et al., 2002). Third, the ATPase activity of full-length CLPB (double-hexamer) is lower than CLPB-NBD alone (S6 Fig). In fact, roughly two thirds of CLPB-NBD particles exhibit a hexameric arrangement. This indicates that the heptamer is not optimized for efficient ATP-hydrolysis. Thus, as a substrate-free state, the heptameric form could avoid necessary ATP consumption in the IMS. Notably, the buffer we used to prepare CLPB complexes was at pH 6.8, which closely resembles that of the IMS (Santo-Domingo and Demaurex, 2012). Therefore, we propose that if the heptameric form is not an in vitro artifact, it could be a resting state of CLPB in mitochondria that could respond to the ATP concentration and the availability of substrates to change its oligomeric states. This interesting hypothesis merits further investigation.”

Both the mammalian Bcs1 (Tang et al., 2020) and yeast Bcs1 (Kater et al., 2020) were reported to assemble into heptameric homo-oligomers exclusively (Rebuttal Figure 2). No other oligomeric states were found.

Rebuttal Figure 2. mBcs1 and yBcs1 assemble into heptameric homo-oligomers exclusively. [Adapted from (Kater et al., 2020; Tang et al., 2020)]

We could not find the bacterial homolog of Bcs1 mentioned by the reviewer. One possible study that the reviewer might have referred to is a preprint (Kwon et al., 2022, Biorxiv, <https://doi.org/10.1101/2022.05.06.490860>). This study reported the structure a bacterial AAA+ hexamer YjoB with unknown function. The authors found that although the overall fold of the YjoB-NTD was partially similar to that of the Bcs1-NTD to a certain extent, they have very low sequence homology, and YjoB is not a membrane protein. Based on the authors’ data, the function of YjoB might be to prevent the aggregation of metabolic enzymes KatA and GapB, which is totally different from Bcs1 translocase. Therefore, we think that it is unclear whether they are functionally counterparts.

The authors stated that the nonamer is non-physiological and no follow-up mutational work was done. Consequently, the value the nonamer structure is unclear and undoubtedly will further clutter the literature.

RE: As mentioned above, the purpose of these domain structures is to provide high-resolution templates for structural interpretation of double-heptamer and double hexamer. This is very common in X-ray crystallography studies. One needs to engineer the protein, sometimes very heavily, to facilitate crystal growth for structural determination. Protein crystals are of course not physiological, which does not mean valuable information could not be extracted from these structures.

In the high-resolution structures of the NBD^E425Q^, we indeed revealed the atomic interactions between the pore-loops of CLPB and the substrate. This interaction pattern is consistent with the independent study by Cupo et al. (2022), and with other typical AAA+ unfoldases/disaggrefases.

* Line 106-107: As a protein disaggregase, CLPB recognizes aggregated protein as native substrate. Only because a Walker B CLPB mutant can bind an endogenous bacterial protein during protein expression does not imply that a substrate complex was obtained. Independent biochemical verification is required. Since mitochondrial CLPB is exclusively found in animal cells, casein may actually be a more physiological substrate mimic.

RE: Generally, AAA+ ATPases translocate/unfold the substrate in a sequence-independent manner, such as Hsp104 (Gates et al., 2017), Cdc48/p97 (Cooney et al., 2019; Twomey et al., 2019). Therefore, Walker B mutation (E to Q) was commonly used for structural purposes. In recent years, cryo-EM studies on different AAA+ proteins have repeatedly found that the introduction of this mutation resulted in a stable binding of co-purified substrate, including ClpX (Fei et al., 2020), ClpB (Yu et al., 2018), Vps4 (Monroe et al., 2017), AFG32L (Puchades et al., 2019), YME1 (Puchades et al., 2019), Rix7 (Kocaman et al., 2022; Lo et al., 2019) and Drg1 (Ma et al., 2022). It is now becoming a common practice in structural studies for capturing substrate-bound state.

We think that in terms of structures there is no difference between a true substrate and a model (or endogenous peptide) substrate, because one would not expect to determine the side-chain identify of the substrates. For examples, the side-chain identities of embedded substrates in the following AAA+ proteins were not determined either (Cooney et al., 2019; Gates et al., 2017; Kocaman et al., 2022; Lo et al., 2019; Ma et al., 2022; Pan et al., 2021; Puchades et al., 2019; Twomey et al., 2019).

Casein (a family of [phosphoproteins](https://en.wikipedia.org/wiki/Phosphoprotein) abundant in milk and dairy products), is a model substrate for the AAA+ ATPases functional study, because of its long C-terminal intrinsic disorder sequence (S337-Q391, 55 amino acids in the C terminus), which can be caught by the pore-loops of AAA+ ATPases. It is not an endogenous substrate of a certain AAA+ ATPase either, and it is in fact secreted outside the cell.

* Assuming that the double-hexamer structure is physiological, is it possible that a substrate is threaded from the NBD end? Since the authors captured an unknown bacterial protein during protein expression, one might expect to see additional mass density that could indicate the direction of the polypeptide bound in the axial channel.

RE: Based on conserved mechanisms of AAA+ ATPases, it is unlikely the substrate is threaded from the distal face of the NBD ring of CLPB.

We did not observe interpretable density outside the channel, nor in the distal face of the NBD ring. In fact, the cryo-EM structure is a computed average from a large number of particles. Even individual particles have extra density outside the channel, it would be averaged out during 3D reconstruction due to its high heterogeneity.

* The authors claim that the ANK domain has no lateral interactions in the double-hexamer/heptamer (c.f. response to reviewers). Yet, Fig 3G, clearly shows lateral contacts that are also described in the text (lines 182-194). The observation that mutating R178 and/or R227 to Glu severely impaired protein disaggregation is very interesting. Both Arg are part of the lateral interactions between neighboring ANK domains but do not abolish the double hexamer/heptamer when mutated. Since R178 or R227 are not involved in mediating an inter-subunit ANK interaction nor can directly interact with substrate for steric reasons, it would argue that the double-heptamer/hexamer must dissociate and therefore is an in vitro artifact.

RE: The reviewer has misunderstood our statement. As we described in the manuscript, there is no lateral interaction within a single hexamer or heptamer. The figures show the head-to-head ANK interactions between two hexamers (vertical interaction) (Figure 3G, Rebuttal Figure 3).

The dimer interface of the ANK domain is mainly mediated by AM1/2 motifs and the insertion. Based on the electrostatic surface potential, two residues (R178 and R227) of one ANK domain point to a highly negatively charged surface patch (E140, E221, D222 and D223) of the opposite ANK domain (Fig 3G, Rebuttal Figure 3). As we mentioned in the manuscript, the interface between two ANK domains is very extensive. We believe that it not that these two residues are not involved in mediating inter-ANK interaction between two hexamers, it is simply that the two single mutations are not sufficient to completely disrupt the higher-order organization.

As we have responded to the first question of this reviewer, we and Cupo et al. both think that the double-hexamer form is more efficient in disaggregase activity. And we stated in the manuscript that “It is possible that a complete disaggregase cycle of CLPB may involve dynamic remodeling of the dimer interface or even disassociation/re-association of hexamers” (Page 13).

Directly quoted here:

“In the double-hexamer, both hexamers are in active conformation with the substrate being threaded through the central pores of their ATPase rings. This raises an interesting question: Whether or not the two hexamers work in a synergistic manner, since a CLPB hexamer already contains all essential structural features of a unfoldase/disaggregase. A conventional model is that the two hexamers work independently, and upon engagement with a protein aggregate the two hexamers do not need to maintain stable association for all six pairs of ANK domains. However, as seen in the structure of the double-hexamer (Fig. 1D), it is also possible that the axial movement of a CLPB subunit in one hexamer during the ATP-hydrolysis cycle could potentially affect another CLPB subunit in the other hexamer through their tightly associated ANK domains. Unfortunately, our attempt to obtain ANK mutants to separate its roles in structural organization and disaggregase activity has failed (Figs 3E and F). It is possible that a complete disaggregase cycle of CLPB may involve dynamic remodeling of the dimer interface or even disassociation/re-association of hexamers.”

* According to MitoCarta 3.0 (1), none of the 20 highest scoring CLPB substrates listed in "S5-HA-Strep_top 20" are localized to the IMS. This includes OPA1 and AGK, which is surprising. The authors proposed that OPA1 is a CLPB substrate that binds to the double-heptamer to induce the structure of the double-hexamer complex with substrate. OPA1 is a protein of 111-KDa and forms large, multi-subunit tubules (2). Even if CLPB recognizes monomeric OPA1 in non-aggregated form, the protein is too large to "fit into" the double-heptamer without dissociating the oligomer.

RE: For any AAA+ hexamers, it is not needed for a structured substrate to be fitted in. CLPB can simply capture a certain flexible terminus or loop on the substrate. Once the stable interaction between the pore-loop and threaded (flexible) peptide is established, CLPB (and other AAA+ proteins) could continuously thread and translocate the peptide, and by doing so to dissolve the aggregates. CLPB substrates do not need to be soluble IMS proteins exclusively. Membrane proteins on the IMM and OMM with domains or (loop) exposed in the IMS could be potential substrates as well.

OPA1 is essential for normal mitochondrial morphology by regulating the equilibrium between mitochondrial fusion and mitochondrial fission. OPA1 locates in the IMM (a single TM helix of L97-113Y) and the majority of OPA1 is in intermembrane space (T114-960K). A long intrinsic disorder sequence (T114-D211, 98 amino acids) is exposed in the intermembrane space (Rebuttal Figure 3).

Rebuttal Figure 3. The model of human OPA1 predicated by AlphaFold (Jumper et al., 2021). The disordered sequence (T114-D211) is shown in red.

* While several studies suggested that HAX1 is a CLPB substrate (3, 4), HAX-1 is not a mitochondrial protein (1, 5). We further note that neither PRKD2 nor HSP27 (1), whose solubility is apparently controlled by HAX1 in mitochondria (4), are mitochondrial proteins. This work is cited by the authors as evidence for a direct HAX1:CLPB interaction.

RE: HAX1 was not explicitly annotated as a mitochondrial protein in MitoCarta 3.0. But this database has classified HAX1 as “mito-interacting” (Rath et al., 2021) (<https://view.officeapps.live.com/op/view.aspx?src=https%3A%2F%2Fpersonal.broadinstitute.org%2Fscalvo%2FMitoCarta3.0%2FHuman.MitoCarta3.0.xls&wdOrigin=BROWSELINK>). In the “HPA_Main_Location_2020 (reliability)” column of the online table (the third sheet, B Human All Genes), it is described as “Mitochondria (Supported)”. Therefore, based on MitoCarta 3.0 (with integrated literatures before 2020), HAX1 is listed as a possible mitochondrial protein.

Fan et al. (2022) employed immunostaining and protease protection assays to show the mitochondrial location of HAX1 (Rebuttal Figure 4) (Fan et al., 2022). Notably, this study was published after the MitoCarta 3.0 update, and these new experiment evidences were thus not included in the MitoCarta 3.0 annotation.

Rebuttal Figure 4. HAX1 is located in the mitochondrial intermembrane space. [Adapted from (Fan et al., 2022)]

We did not discuss either HSP27 or PRKD2 in our manuscript. Fan et al. reported that HAX1-deletion PLB-985 cells exhibit dramatically reduced expression of HSP27. Although HSP27 is a known cytosolic protein, the authors also showed that HSP27 could be detected in mitochondrial fractions. They found that HAX1 might be involved in maintaining the stability of the phosphorylated, soluble form of HSP27. In the absence of HAX1 and CLPB, the unphosphorylated, insoluble form of HSP27 would accumulate in the mitochondrial lysates (Rebuttal Figure 5) (Fan et al., 2022). These findings indicate that HSP27 is likely a protein of multiple cellular location.

Rebuttal Figure 5. HAX1 regulates phosphorylation and solubility of HSP27 in mitochondria. [Adapted from (Fan et al., 2022)]

As for the HAX1: CLPB interaction, Fan et al., have done several experiments to confirm that HAX1 is an interaction partner and substrate of CLPB (Fan et al., 2022). Firstly, they identified CLPB as one of the most prominent binding partners of HAX1 by transfecting FLAG-tagged HAX1 into HEK293T cells and performing IP experiments and subsequent MS identification. Secondly, they confirmed the mutual interaction between HAX1 and CLPB by bidirectional co-IP experiments (Rebuttal Figure 6). Thirdly, they mapped the interaction regions of CLPB and HAX1, residues 126-136 of HAX1 and ANK domain of CLPB (Rebuttal Figure 6). These data clearly demonstrated that HAX1 is a direct binder of CLPB.

Rebuttal Figure 6. HAX1 interacts with CLPB. [Adapted from (Fan et al., 2022)]

* Fig. S2A and S2E: The SEC chromatogram shows two peaks for CLPB-AMPPNP and CLPBiso1-AMPPNP of near equal height (S2E). What is the nature of the second species consisting of fractions: 16-22, which were not included in the SDS-PAGE gel?

RE: Our CLPB construct contains a 6X-HIS-SUMO tag in the N-terminus. Proteins were purified through ammonium sulfate precipitation, followed by an ion-exchange chromatography (Mono Q). TEV protease was used to remove the HIS-SUMO tag. This second peak is composed of TEV enzyme and cleaved HIS-SUMO tag.

* Line 305: "potentially thread the substrate in both directions, similar as bacterial ClpX and ClpA". This is a misleading statement. Unlike CLPB, bacterial ClpX and ClpA are stably bound to the ClpP peptidase that confers unidirectional substrate-threading from an N-to-C direction. However, this may not be the case for CLPPB.

RE: The reviewers’ statement is incorrect. Numerous studies have showed that ClpXP and ClpAP could translocate and degrade substrates in either C-to-N or N-to-C direction (see the review by Olivares, Baker and Sauer, 2018, Annual review of Physiology). The direction would be dependent on the location of specific degrons. For example, the SsrA tag is added to the C-terminus of a substrate protein by the tmRNA system, and ClpXP would translocate the SsrA-bearing substrate in a C-to-N manner in the cells.

Responses to reviewers' queries:

* (Rev 1-3): Obviously there are lateral interactions between ANK domains of neighboring subunits (c.f. Fig 3G). It is unclear what is implied by stating that "[the ANK domain] has no lateral interaction with the adjacent ANK domains within the hexamer/heptamer.

RE: As mentioned above, there is no lateral interaction within a single hexamer or heptamer. The figures show the head-to-head interactions between two ANK domains from two hexamers (vertical interaction) (Figure 3G).

* (Rev. 2): ATP, AMP-PNP, and ATPgS are quite different and sometimes promote off-pathway conformations. They also perform differently is substrate interaction. Structurally they are quite different as well, which can be seen at atomic resolution but is beyond the resolution of the present cryoEM reconstructions. The rebuttal figure 2 is unsatisfactory because Hsp104 is an entirely different protein. The authors should perform this experiment with their CLPB protein used for structure analysis.

RE: The rebuttal figure 2 (Revision 1) is our response to why we have changed the nucleotide from AMPPNP to ATPγS for the analysis of oligomeric states. Based on published studies of Hsp104 (Gates et al., 2017; Weaver et al., 2017), ATPγS is the best to maximize the binding fraction of Hsp104 to casein. For this reason, we have chosen to use ATPγS to maximize the binding of casein to the WT CLPB complex.

The recent study by Cupo et al. has examined the binding of casein to CLPB in the presence different nucleotides. They found that ATPγS could best promote the binding of casein (Rebuttal Figure 7).

We have cited this data from Cupo et al. in our revision.

Rebuttal Figure 7. FITC-casein binding analysis, measured by fluorescence polarization in the presence of no nucleotide (APO; grey), AMP-PNP (blue), ADP (red), ATP(green), and ATPγS (black)..[Adapted from (Cupo et al., 2022)]

* (Rev. 3): "a very recent NMR study on Hsp104 has identified a substrate-binding groove in the NTD to recognize exposed hydrophobic stretches in substrates of Hsp104 (Harari et al., 2022)). The ANK domain may work in a similar fashion to bind to exposed hydrophobic sequences of the substrate." The NTD of Hsp104 and CLPB are neither related nor similar in structure. Inferring any mechanistic insights by direct comparison is wrong.

RE: Both of them are involved in substrate binding, and their substrates (unfolded or misfold segments from a protein) could be hydrophobic and should be aggregation-prone. We are not inferring any mechanistic insights directly from Hsp104. It is simply a generalization of possible role of the ANK of CLPB, because NTDs of many AAA+ hexamers, although structurally and functionally distinct, are all involved in substrate binding.

* (Rev 3): "a study employed proteinase K assay on isolated mitochondria and showed that the degradation of HAX1 (CLPB as well) requires the disruption of the outer membrane (Fan et al., 2022). Therefore, we think that it could be a mis-annotation of MitoCarta 3.0." I do not doubt that HAX-1 may mimic a substrate (as casein does), but that does not mean this interaction is physiological and needs to be taken with caution.

RE: Thank you for this comment. We totally agree, and have revised the manuscript to change the tone related to the description of CLPB-HAX1 interaction.

1. Rath S, Sharma R, Gupta R, Ast T, Chan C, Durham TJ, et al. MitoCarta3.0: an updated mitochondrial proteome now with sub-organelle localization and pathway annotations. Nucleic Acids Res. 2021;49(D1):D1541-d7.

2. Zhang D, Zhang Y, Ma J, Zhu C, Niu T, Chen W, et al. Cryo-EM structures of S-OPA1 reveal its interactions with membrane and changes upon nucleotide binding. Elife. 2020;9.

3. Chen X, Glytsou C, Zhou H, Narang S, Reyna DE, Lopez A, et al. Targeting mitochondrial structure sensitizes acute myeloid leukemia to venetoclax treatment. Cancer Discov. 2019;9(7):890-909.

4. Fan Y, Murgia M, Linder MI, Mizoguchi Y, Wang C, Łyszkiewicz M, et al. HAX1-dependent control of mitochondrial proteostasis governs neutrophil granulocyte differentiation. J Clin Invest. 2022;132:e153153.

5. Jeyaraju DV, Cisbani G, De Brito OM, Koonin EV, Pellegrini L. Hax1 lacks BH modules and is peripherally associated to heavy membranes: implications for Omi/HtrA2 and PARL activity in the regulation of mitochondrial stress and apoptosis. Cell Death Differ. 2009;16:1622-9.

**Reviewer #4 (Christopher Hill, signs review):**

The revised manuscript seems acceptable for publication. However, it would benefit substantially from copying editing to improve the use of English.

RE: We thank Reviewer #1 for his expert opinion and recommendation to publish our manuscript.

**Reference**

Chen, X., Glytsou, C., Zhou, H., Narang, S., Reyna, D.E., Lopez, A., Sakellaropoulos, T., Gong, Y., Kloetgen, A., Yap, Y.S.*, et al.* (2019). Targeting Mitochondrial Structure Sensitizes Acute Myeloid Leukemia to Venetoclax Treatment. Cancer Discov *9*, 890-909.

Cooney, I., Han, H., Stewart, M.G., Carson, R.H., Hansen, D.T., Iwasa, J.H., Price, J.C., Hill, C.P., and Shen, P.S. (2019). Structure of the Cdc48 segregase in the act of unfolding an authentic substrate. Science *365*, 502-505.

Cupo, R.R., Rizo, A.N., Braun, G.A., Tse, E., Chuang, E., Gupta, K., Southworth, D.R., and Shorter, J. (2022). Unique structural features govern the activity of a human mitochondrial AAA+ disaggregase, Skd3. Cell Rep *40*, 111408.

Cupo, R.R., and Shorter, J. (2020). Skd3 (human ClpB) is a potent mitochondrial protein disaggregase that is inactivated by 3-methylglutaconic aciduria-linked mutations. Elife *9*.

Fan, Y., Murgia, M., Linder, M.I., Mizoguchi, Y., Wang, C., Lyszkiewicz, M., Zietara, N., Liu, Y., Frenz, S., Sciuccati, G.*, et al.* (2022). HAX1-dependent control of mitochondrial proteostasis governs neutrophil granulocyte differentiation. J Clin Invest *132*.

Fei, X., Bell, T.A., Jenni, S., Stinson, B.M., Baker, T.A., Harrison, S.C., and Sauer, R.T. (2020). Structures of the ATP-fueled ClpXP proteolytic machine bound to protein substrate. Elife *9*.

Gates, S.N., Yokom, A.L., Lin, J., Jackrel, M.E., Rizo, A.N., Kendsersky, N.M., Buell, C.E., Sweeny, E.A., Mack, K.L., Chuang, E.*, et al.* (2017). Ratchet-like polypeptide translocation mechanism of the AAA+ disaggregase Hsp104. Science *357*, 273-279.

Hoffmann, F.T., Kim, M., Beh, L.Y., Wang, J., Vo, P.L.H., Gelsinger, D.R., George, J.T., Acree, C., Mohabir, J.T., Fernandez, I.S.*, et al.* (2022). Selective TnsC recruitment enhances the fidelity of RNA-guided transposition. Nature *609*, 384-393.

Jumper, J., Evans, R., Pritzel, A., Green, T., Figurnov, M., Ronneberger, O., Tunyasuvunakool, K., Bates, R., Zidek, A., Potapenko, A.*, et al.* (2021). Highly accurate protein structure prediction with AlphaFold. Nature *596*, 583-589.

Kater, L., Wagener, N., Berninghausen, O., Becker, T., Neupert, W., and Beckmann, R. (2020). Structure of the Bcs1 AAA-ATPase suggests an airlock-like translocation mechanism for folded proteins. Nat Struct Mol Biol *27*, 142-149.

Kim, G., Lee, S.G., Han, S.S., Jung, J., Jeong, H.S., Hyun, J.K., Rhee, D.K., Kim, H.M., and Lee, S. (2020). ClpL is a functionally active tetradecameric AAA plus chaperone, distinct from hexameric/dodecameric ones. Faseb J *34*, 14353-14370.

Kocaman, S., Lo, Y.-H., Krahn, J.M., Sobhany, M., Dandey, V.P., Petrovich, M.L., Etigunta, S.K., Williams, J.G., Deterding, L.J., Borgnia, M.J.*, et al.* (2022). Communication network within the essential AAA-ATPase Rix7 drives ribosome assembly. PNAS Nexus.

Lo, Y.H., Sobhany, M., Hsu, A.L., Ford, B.L., Krahn, J.M., Borgnia, M.J., and Stanley, R.E. (2019). Cryo-EM structure of the essential ribosome assembly AAA-ATPase Rix7. Nat Commun *10*, 513.

Ma, C., Wu, D., Chen, Q., and Gao, N. (2022). Structural dynamics of AAA + ATPase Drg1 and mechanism of benzo-diazaborine inhibition. Nat Commun *13*, 6765.

Miyata, T., Yamada, K., Iwasaki, H., Shinagawa, H., Morikawa, K., and Mayanagi, K. (2000). Two different oligomeric states of the RuvB branch migration motor protein as revealed by electron microscopy. J Struct Biol *131*, 83-89.

Monroe, N., Han, H., Shen, P.S., Sundquist, W.I., and Hill, C.P. (2017). Structural basis of protein translocation by the Vps4-Vta1 AAA ATPase. Elife *6*.

Pan, M., Yu, Y., Ai, H., Zheng, Q., Xie, Y., Liu, L., and Zhao, M. (2021). Mechanistic insight into substrate processing and allosteric inhibition of human p97. Nat Struct Mol Biol *28*, 614-625.

Puchades, C., Ding, B., Song, A., Wiseman, R.L., Lander, G.C., and Glynn, S.E. (2019). Unique Structural Features of the Mitochondrial AAA+ Protease AFG3L2 Reveal the Molecular Basis for Activity in Health and Disease. Mol Cell *75*, 1073-1085 e1076.

Rath, S., Sharma, R., Gupta, R., Ast, T., Chan, C., Durham, T.J., Goodman, R.P., Grabarek, Z., Haas, M.E., Hung, W.H.W.*, et al.* (2021). MitoCarta3.0: an updated mitochondrial proteome now with sub-organelle localization and pathway annotations. Nucleic Acids Res *49*, D1541-D1547.

Santo-Domingo, J., and Demaurex, N. (2012). Perspectives on: SGP symposium on mitochondrial physiology and medicine: the renaissance of mitochondrial pH. J Gen Physiol *139*, 415-423.

Santosh, V., Musayev, F.N., Jaiswal, R., Zarate-Perez, F., Vandewinkel, B., Dierckx, C., Endicott, M., Sharifi, K., Dryden, K., Henckaerts, E.*, et al.* (2020). The Cryo-EM structure of AAV2 Rep68 in complex with ssDNA reveals a malleable AAA(+) machine that can switch between oligomeric states. Nucleic Acids Research *48*, 12983-12999.

Tang, W.K., Borgnia, M.J., Hsu, A.L., Esser, L., Fox, T., de Val, N., and Xia, D. (2020). Structures of AAA protein translocase Bcs1 suggest translocation mechanism of a folded protein. Nat Struct Mol Biol *27*, 202-209.

Twomey, E.C., Ji, Z., Wales, T.E., Bodnar, N.O., Ficarro, S.B., Marto, J.A., Engen, J.R., and Rapoport, T.A. (2019). Substrate processing by the Cdc48 ATPase complex is initiated by ubiquitin unfolding. Science *365*.

Weaver, C.L., Duran, E.C., Mack, K.L., Lin, J., Jackrel, M.E., Sweeny, E.A., Shorter, J., and Lucius, A.L. (2017). Avidity for Polypeptide Binding by Nucleotide-Bound Hsp104 Structures. Biochemistry *56*, 2071-2075.

Yu, H., Lupoli, T.J., Kovach, A., Meng, X., Zhao, G., Nathan, C.F., and Li, H. (2018). ATP hydrolysis-coupled peptide translocation mechanism of Mycobacterium tuberculosis ClpB. Proc Natl Acad Sci U S A *115*, E9560-E9569.

Yu, X., VanLoock, M.S., Poplawski, A., Kelman, Z., Xiang, T., Tye, B.K., and Egelman, E.H. (2002). The Methanobacterium thermoautotrophicum MCM protein can form heptameric rings. Embo Rep *3*, 792-797.
